# Implementation of a Web-Based Outpatient Asynchronous Consultation Service: Mixed Methods Study

**DOI:** 10.2196/48092

**Published:** 2024-06-04

**Authors:** Magdalena Rzewuska Díaz, Louise Locock, Andrew Keen, Mike Melvin, Anthony Myhill, Craig Ramsay

**Affiliations:** 1 Health Services Research Unit University of Aberdeen Aberdeen United Kingdom; 2 NHS Grampian Grampian Aberdeen United Kingdom; 3 Public Research Partner University of Aberdeen Aberdeen United Kingdom

**Keywords:** outpatient care, teleconsultation, asynchronous communication, implementation, mixed methods research, qualitative research, hospital data

## Abstract

**Background:**

Asynchronous outpatient patient-to-provider communication is expanding in UK health care, requiring evaluation. During the pandemic, Aberdeen Royal Infirmary in Scotland expanded its outpatient asynchronous consultation service from dermatology (deployed in May 2020) to gastroenterology and pain management clinics.

**Objective:**

We conducted a mixed methods study using staff, patient, and public perspectives and National Health Service (NHS) numerical data to obtain a rounded picture of innovation as it happened.

**Methods:**

Focus groups (3 web-based and 1 face-to-face; n=22) assessed public readiness for this service, and 14 interviews with staff focused on service design and delivery. The service’s effects were examined using NHS Grampian service use data, a patient satisfaction survey (n=66), and 6 follow-up patient interviews. Survey responses were descriptively analyzed. Demographics, acceptability, nonattendance rates, and appointment outcomes of users were compared across levels of area deprivation in which they live and medical specialties. Interviews and focus groups underwent theory-informed thematic analysis.

**Results:**

Staff anticipated a simple technical system transfer from dermatology to other receptive medical specialties, but despite a favorable setting and organizational assistance, it was complicated. Key implementation difficulties included pandemic-induced technical integration delays, misalignment with existing administrative processes, and discontinuity in project management. The pain management clinic began asynchronous consultations (*digital appointments*) in December 2021, followed by the gastroenterology clinic in February 2022. Staff quickly learned how to explain and use this service. It was thought to function better for pain management as it fitted preexisting practices. From May to September 2022, the dermatology (adult and pediatric), gastroenterology, and pain management clinics offered 1709 appointments to a range of patients (n=1417). Digital appointments reduced travel by an estimated 44,712 miles (~71,956.81 km) compared to the face-to-face mode. The deprivation profile of people who chose to use this service closely mirrored that of NHS Grampian’s population overall. There was no evidence that deprivation impacted whether digital appointment users subsequently received treatment. Only 18% (12/66) of survey respondents were unhappy or very unhappy with being offered a digital appointment. The benefits mentioned included better access, convenience, decreased travel and waiting time, information sharing, and clinical flexibility. Overall, patients, the public, and staff recognized its potential as an NHS service but highlighted informed choice and flexibility. Better communication—including the use of the term *assessment* instead of *appointment*—may increase patient acceptance.

**Conclusions:**

Asynchronous pain management and gastroenterology consultations are viable and acceptable. Implementing this service is easiest when existing administrative processes face minimal disruption, although continuous support is needed. This study can inform practical strategies for supporting staff in adopting asynchronous consultations (eg, preparing for nonlinearity and addressing task issues). Patients need clear explanations and access to technical support, along with varied consultation options, to ensure digital inclusion.

## Introduction

### Background

In recent decades, health care organizations have adopted innovative remote patient-to-provider communication methods such as video, instant messaging, and email. The nature of the COVID-19 pandemic caused extraordinary growth in their use, which relieved health system strains by allowing patients to obtain primary and specialized care without an in-person appointment [1,2]. As these new forms of patient-to-provider communication are expected to become a part of delivering health care, valuable lessons can be learned from this accelerated uptake under emergency measures [3].

Asynchronous consultations, where health care staff and patients do not need to be available at the same time, are a potential alternative to synchronous methods (in real time), such as traditional in-person meetings, phone calls, and videoconferences. They have been widely tested in primary care [4-8] but are increasingly used in secondary care outpatient services, particularly for highly visible symptoms such as dermatological problems [9,10] or epileptic seizures [11]. Typically, outpatient asynchronous approaches involve patients answering a number of specialty-specific questions about their health over the web at times that suit them, usually within a window of approximately a week (responses may include photographs or video footage). This completed form is then reviewed by the outpatient clinic, and they can request further information over a similar period before deciding on the most suitable outcome (eg, examinations, treatments, face-to-face consultations, or outpatient discharge).

Asynchronous consultations provide a new health care model, unlike videoconferencing. A systematic review found that outpatient asynchronous consultations may produce equivalent outcomes to in-person treatment and lower health care expenses for certain conditions [12]. The pandemic has increased the acceptability of remote consultations, but technical, educational, infrastructure, legal, and economic challenges must be solved for them to be sustainable and scalable beyond the pandemic [13]. Because of this, emphasis has been placed on determining the resources and approaches required to effectively implement the technology required to deliver asynchronous consultations (by assessing how they are adopted) and understanding their effects on staff and patients [3,12,14,15].

### Objectives

A nationwide case study by Wherton et al [8] indicated that National Health Service (NHS) boards in Scotland expanded video consultations before and after the epidemic. The NHS Grampian board serves a population of nearly 600,000 in urban, remote, and rural locations across a large geographic area. It also provides some specialist services to other boards in the north of Scotland, including the island communities of Shetland and Orkney. Similar to other boards in Scotland but to a greater extent, NHS Grampian substantially increased its use of video consultations [8]. The Department of Dermatology at Aberdeen Royal Infirmary introduced asynchronous consultations as part of a multiboard, nationally led innovation project in May 2020. Due to the pandemic, the organization expanded this new service to the gastroenterology and pain management clinics. The dermatology system provided a template and contextual insights for rolling out this new service. This study examined staff, patient, and public perspectives and NHS quantitative data to provide a complete picture of the outpatient asynchronous consultation service adoption.

## Methods

### Study Context

The list of parties involved in developing and implementing new pathways is shown in Textbox 1. The NHS Grampian team approached the Health Services Research Unit for help with assessing this implementation’s effects. An implementation researcher (MRD), an expert qualitative social science researcher (LL), a service improvement researcher (CR), NHS Grampian’s clinical lead for innovation (AK), a service evaluation lead (KM), and 2 public research partners knowledgeable in digital skills assistance (MM and AM) formed the study team. The lay partners participated in project conceptualization and all research stages as coinvestigators (from design to delivery and dissemination).

**A list of adopters of the newly implemented pathways (gastroenterology and pain management) of asynchronous consultations.**

**Stakeholders and responsibilities**

- Asynchronous coordinator: supporting the administrative process surrounding the onboarding of patients and logging issues with the third-party supplier; assisting with troubleshooting
- Clinical leads: defining pathway question sets associated with their specialty
- Clinic coordinators: booking patients into asynchronous consultations and answering some onboarding queries (although this was most likely done by the asynchronous coordinator)
- Clinicians: reviewing the information provided by the patients within the asynchronous consultations and issuing an outcome via the software to the patient
- eHealth: involved in the technical configuration of appointment messaging and outgoing, and onward delivery to appropriate National Health Service (NHS) Grampian electronic patient records; calls may also be logged on the IT service desk, but these are mainly resolved by the third-party supplier, National Services Scotland (NSS), or administrative support teams involved in supporting the system within their departments
- Information governance: ensuring that the project provides the appropriate assurances to enable an NHS Grampian data protection officer Caldicott Guardian to approve required paperwork associated with the system
- IT security: ensuring that the project provides the appropriate assurances to enable the IT security officer to approve required paperwork associated with the system
- NSS and NHS Education for Scotland: involved in technical configuration and delivery of messaging between NHS Grampian and third-party supplier systems and technical troubleshooting
- Outpatient services management: overall responsibility for the patient information leaflets that give patients information about the asynchronous consultations
- Project team: managing the product rollout within the pain management and gastroenterology clinics, coordinating all staff involved to ensure that all prerequisites are met, facilitating a smooth transition to the deployment phase; managing handover to business-as-usual teams
- Service management (gastroenterology only): helping ensure that the process is embedded within gastroenterology
- Storm ID: supplier of the software; enhancing the software and troubleshooting issues

### The Asynchronous Consultation System

**Figure 1:**
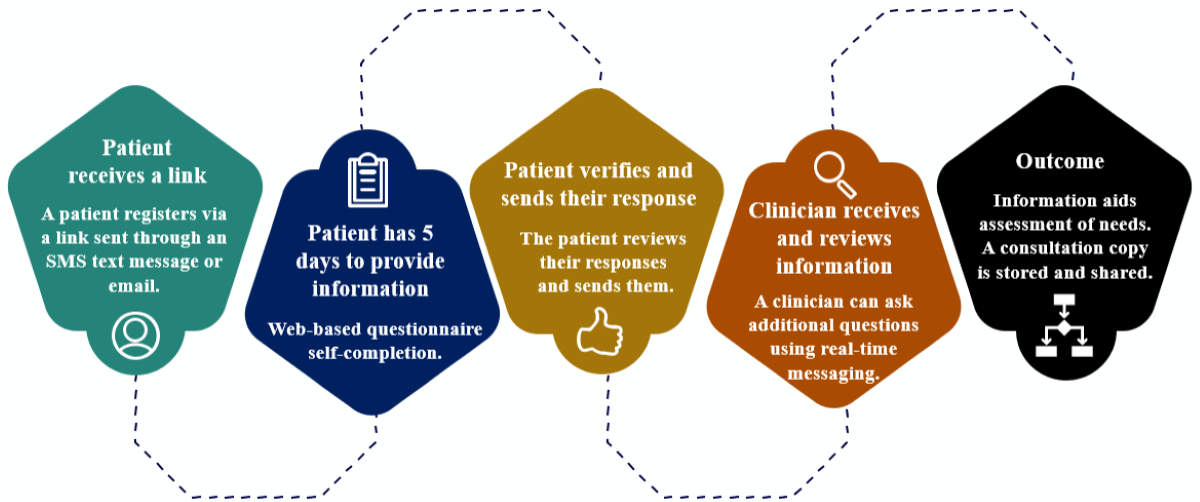
Digital appointments: an asynchronous consultation process within the National Health Service Grampian.

This service is for routine appointments only. An outpatient team vets returning or new patients referred by primary care physicians to assess suitability for an asynchronous consultation (that NHS Grampian calls *digital appointments*). After reaching the top of the waiting list, a patient receives an information pamphlet explaining a digital appointment and a letter encouraging them to call the operational team to book. If the patient declines, the booking staff offer a synchronous alternative. An appointment is generated in TrakCare, a patient management system, which then triggers the invitation to the patient to register for the digital system. After registration, the patient receives a link to a web-based inquiry form (a web-based questionnaire) with 5 days to complete before the scheduled date (Figure 1). Sample areas of questioning in the pain management pathway include experience of pain, symptom descriptions and improvement level, adverse effects, pain medication, mood, ability to undertake normal work, sleep, enjoyment of life, and any other relevant experiences. Sample areas of questioning in the gastroenterology pathway include description of symptoms, bowel movements and stool, experience of abdominal pain and bloating, weight loss, medication taken, ability to perform normal work and daily tasks, enjoyment of life, and concerns. Patients register, answer questions, check, and submit. After analyzing the information and maybe asking further questions through real-time messaging, a clinician determines whether to provide remote advice and treatment, schedule a face-to-face visit, or discharge. The patient, outpatient clinical team, and general practitioner receive a PDF appointment summary (via the NHS email address of a clinician system user) stored in a document repository (Skystore). Delivery is via Storm ID (third party), and Lenus is the platform used.

### Study Design

We deployed a mixed method study design with a multistage purposeful sampling strategy [16]. It included public focus groups about public readiness for the new service (3 web-based and 1 face-to-face), interviews with staff about their experiences with designing and implementing the service; and an assessment of service uptake conducted through analysis of the service use data and patient experience (including a patient satisfaction survey and follow-up patient interviews about their experiences with receiving the service). Qualitative study, including interviews and focus groups, ran from April 2021 to July 2022. The NHS service evaluation covered a satisfaction survey from January 2022 to September 2022 and analysis of NHS Grampian service usage data between May 2020 and September 2022.

The primary focus was on the newly implemented pathways—gastroenterology and pain management—whereas adult dermatology served effectively to provide context, helping illustrate the transferability from one medical specialty to another in a real-world scenario. Focus groups with members of the public and staff interviews covered all 3 medical specialties. The NHS Grampian data on service use covered 4 medical specialties: pain management, gastroenterology, and adult and pediatric dermatology. The patient satisfaction survey, conducted by NHS Grampian, was confined by our ethical clearance to prospective evaluations of the newly implemented pathways as dermatology was assessed independently. All participants in the satisfaction survey who were happy to be contacted by a researcher were eligible for the interviews.

Overall, the study was designed to answer six research questions:

1. How accepting and prepared is the public for asynchronous consultations?
2. What are clinical and administrative staff experiences of and attitudes toward asynchronous consultation in secondary care?
3. Is asynchronous consultation sustainable beyond the immediate context of COVID-19?
4. How do patients feel about the quality of asynchronous consultations and their effect on their relationship with health care professionals?
5. How do asynchronous consultations affect health inequalities in terms of access?
6. What is the impact of an asynchronous consultation model on NHS performance?

### Ethical Considerations

The NHS service evaluation component (satisfaction survey and NHS Grampian service use data analysis) led by the NHS Grampian did not require ethical review. Ethics approval for the qualitative study was granted by the London–Bloomsbury Research Ethics Committee (reference 21/PR/0051). Active informed verbal or written consent was obtained from each qualitative study participant individually. Potential participants had at least 24 hours to consider whether or not they would like to participate in the research and were free to withdraw at any time and without giving a reason. As compensation for their time and effort, the public focus group participants were offered a £15 (approximately US $19.5) retail-gift voucher, but staff and patient interview participants were not compensated for their time. Qualitative data were deidentified before analyzing.

### Focus Groups About Public Readiness

#### Recruitment and Data Collection

We recruited members of the public for participation in focus groups, targeting individuals at risk of digital exclusion, including low-income people, older people, people with disabilities, and those who assist them. Our 2 public coinvestigators (MM and AM) who work with community voluntary sector organizations contacted members of the public to invite them to participate. In total, 3 web-based (using Microsoft Teams platform) focus groups and 1 face-to-face focus group of 4 to 7 participants were held, with 22 adult participants in total (n=8, 36% men and n=14, 64% women; n=6, 27% aged 20-40 years; n=5, 23% aged 41-64 years; and n=11, 50% aged 65 years). The asynchronous consultation service used by the dermatology clinic was explained and demonstrated to participants at the start of the focus groups. In addition, the group was informed about what new medical specialties this service would be expanded to, highlighting its potential applicability across various populations. The facilitator (MRD) used a semistructured topic guide (Multimedia Appendix 1, item A).

#### Data Analysis

Focus groups were video or audio recorded and professionally transcribed, checked for accuracy, deidentified, and coded using the NVivo software (version 10; Lumivero). The technology acceptance model [17]—one of the most influential technology acceptance models, which proposes that perceived ease of use and usefulness of a technological tool determine the extent of user acceptance—informed the analysis. Analysis began alongside data collection, with ideas from early analysis informing later data collection in an iterative process. Analysis of transcripts commenced with familiarization conducted by 2 experienced qualitative researchers (MRD and LL) followed by open coding using a mixture of inductive and theory-informed codes. This generated a coding framework, which then one researcher (MRD) systemically applied to all data. The themes identified from the focus groups helped inform later qualitative stages (see the following sections).

### Staff Interviews About Design and Implementation

#### Recruitment and Data Collection

NHS Grampian partners contacted staff members who expressed interest by contacting the project researcher. Staff were interviewed over the web (Microsoft Teams platform) using topic guides (Multimedia Appendix 1, item B) to discuss dermatology routes retrospectively and pain management and gastrointestinal routes prospectively. From the service adopters described in Textbox 1 (gastroenterology and pain management pathways), 8 people, including clinical leads, service or administration leads, and technological leads or experts (including a developer, technical integration lead, and digital health lead), were interviewed at the preimplementation project stage (June to November 2021), of whom 5 (63%) had experience with either the dermatology system alone or dermatology and the newly implemented pathways. At this stage, we additionally interviewed a dermatology clinician, totaling 9 individuals interviewed at the preimplementation stage. A total of 5 adopters of the newly implemented pathways (n=2, 40% interviewed at the preimplementation stage) were interviewed at the postimplementation stage (March to June 2022), including 2 (40%) clinical leads, 1 (20%) service or administration lead, and project management leads or experts (including an evaluation lead and a project manager).

#### Data Analysis

The approach was the same as for focus group analysis, but this time the coding framework was informed by the evidence-based conceptual model by Greenhalgh et al [18] for evaluating factors affecting diffusion, dissemination, and implementation of innovations in health care delivery and organization.

### Assessment of Service Uptake by Patients

#### Assessment of Routinely Collected Data

The NHS Grampian team examined service use data, including demographics of new system users (age, postcode, and sex), acceptance of service throughout routes (numbers completed, expressly declined, and not accepted for other reasons), nonattendance rates, and appointment results. To assess health inequality, we evaluated rate distribution across the Scottish Index of Multiple Deprivation (SIMD) [19], which ranks every small area in NHS Grampian from *most deprived* (lowest quintile of the SIMD) to *least deprived* (highest quintile of the SIMD). The data were based on hospital data collected between May 2020 and September 2022 (in this, new pathways data spanned the period from January to September 2022) and an audit carried out by administrative staff between February and July 2022. When feasible, a chi-square test was conducted to calculate percentage differences, setting the significance level at *P*>.05.

#### Patient Experience

All gastroenterology and pain management patients who were offered the new service were given the chance to complete a brief web-based satisfaction survey (Multimedia Appendix 1, items D-F). Collected data were analyzed descriptively. Patients who completed a survey and checked a box to be contacted by the project researcher were eligible for a follow-up interview. The NHS Grampian team research partners gave the details of consenting, eligible participants to the study researcher. The researcher (MRD) contacted participants via email or phone call and sent reminders at 2 weeks. Semistructured interviews were conducted over the web (Microsoft Teams platform) or by phone to better understand their experiences (see Multimedia Appendix 1, item C, for the patient interview topic guide) and analyzed in the same way as the focus group transcripts. From March to July 2022, a total of 6 pain management patients were interviewed, of whom 4 (67%) completed their appointments and 2 (33%) did not. Table S1 in Multimedia Appendix 2 describes the interview participants. Only 2 gastroenterology patients consented to be contacted by the researchers, but neither responded.

## Results

### Focus Groups About Public Readiness

#### COVID-19’s Impact on the Digital Revolution

Participants described how the COVID-19 pandemic significantly affected the population’s digital technology habits, including remote consultations, and has sped up many valuable previously hard-to-imagine digital innovations. They aired concerns about how this may change the fabric of society, adding to the problem of technology addiction and diminishing human interactions.

#### Attitudes Toward Asynchronous Consultations

Some participants described having personal, satisfactory experiences using remote communication (emails and phone and video calls) with health care providers. Most participants were very positive about the new asynchronous consultation system. Even those who expressed a clear personal preference for remote synchronous and face-to-face consultations could see how the service could be useful for some aspects of care, such as triage, monitoring the health care needs of people with long-term conditions, or reaching people who have difficulty traveling to hospital.

#### Relative Advantages and Disadvantages of Asynchronous Consultations

Many possible benefits were cited regarding access, efficiency, time and travel saved, and more flexibility for clinicians, as well as reminders that we should not make assumptions about who would find asynchronous consultations hard to use. Downsides were also aired, particularly for those for whom English is a second language, people without a good internet connection or adequate devices, people with conditions that need physical examination or symptoms that are difficult to describe in words, and people with cognitive or developmental disabilities; the downsides included risks of urgent issues being missed, loss of human contact and empathy, fragmentation of care, and the potential that the length of time one must wait for a reply could create anxiety (although accepting it might still be quicker than waiting for a face-to-face appointment).

#### Practical Barriers to and Facilitators of Using the Asynchronous Service

Participants noted that the practical barriers, such as the lack of equipment and skills, could be mitigated with the help of community-funded programs or by setting up adequate and safe spaces in the community or providing an alternative method of communication (such as telephone). Patients’ fears regarding data privacy and confidentiality would need to be allayed, the group explained, as these issues would otherwise serve as a barrier.

#### Compatibility With Needs

As the new service might not work for everyone, participants stressed the importance of being mindful of personal preferences (eg, for communication format), tailoring the system to end users’ needs, and the service remaining optional. Most participants wondered whether being routed to an asynchronous system might make people feel fobbed off or less valued; another person noted that those who could not use it might feel less valued or like second-class citizens. The underlying concern was that digital exclusion and opting out could exacerbate health inequalities.

Further details on findings under the developed themes can be found in Multimedia Appendix 2.

### Staff Interviews About Design and Implementation

Findings from staff interviews are presented drawing on components of the conceptual model by Greenhalgh et al [18] for the diffusion and implementation of innovations. Further details on findings under the developed themes and on implementation setbacks specifically can be found in Tables S2 and S3 in Multimedia Appendix 2, respectively.

#### Characteristics of the Innovation Itself

Staff involved in the implementation of asynchronous consultations were motivated to roll out the innovation by their assessment that the new system could give clinicians more flexibility, give patients more convenience, enable access to the right care for the right people at the right time (triage or assessment), and improve information sharing (among a patient, an outpatient clinic, and general practice). Over time, those benefits began to manifest. For example, staff noted that, at first, going over a digital appointment response was not much faster than a phone call but, later, it became quicker, taking 15 minutes instead of 30 minutes. Digital exclusion and adding an extra step to the care pathway were among the early concerns that adopters wished to evaluate. The most mentioned concern was a loss of human contact, the risk of which staff felt was reduced by using real-time messaging functions with new patients or creating a pathway where patients are already engaged with care providers. Overall, there was alignment with public opinions, particularly regarding the use for triage and main concerns about digital exclusion and the loss of human contact:

You know, we’ve got a better understanding of the impact of pain and their mood and their function, and how much it’s interrupting daily activities, and that’s not information that we would have had before we would have had to gained all the information during the first appointment.

Clinical lead 3

[in pain pathways] Patients are already engaged following a face-to-face appointment with the nursing teams prior to the async appointments. This provides a human touch and reassurance that patients’ issues are known and will be considered, listened to.

Project management lead or expert 1

It was generally accepted that the innovation might be somewhat changed to satisfy different needs except when the product design and deployment are controlled centrally as opposed to locally (as a part of the national pilot or scale-up project). The new pathways’ clinical leads determined the type of information gathered, the questions to be asked during the consultation, and the place of the digital appointment in the patient experience, which mirrors the public’s expectations for the service to be tailored to the needs of the end users. Clinical leads described 2 examples of how the system’s function evolved according to needs after it went live (eg, the pain management clinic started to use the system to manage medication use) and continued proposing new modifications (eg, a clinical lead in gastroenterology described plans for being able to share educational materials with patients).

The developers of the newly implemented pathways viewed practical demonstrations of the dermatology system as helpful (especially pathway design and patient booking process). For instance, adopters of the new pathway tried to draw lessons from the fact that the dermatology pathway was initially too disjointed and complex for patients. Still, there was a perceived lack of space and time to try out the new systems before they went live, but they recognized that the new process could only be tested once technically integrated with the system.

After launching the service, it became evident that the new service created additional tasks for staff. Staff described additional work tasks related to appointment booking and management process, such as manual record keeping, chasing up dermatology patients, and having to phone patients to offer an appointment. In general, clinicians’ manual selection of patients from waiting lists or administrative staff’s selection of patients out of order and cold calls to patients were seen as ineffective. A clinical lead explained how, initially, at their end, there was also a lot of paperwork involved. However, later, staff’s familiarization with the technology and growing confidence promoted more efficient use of the system (eg, using a filter function to search waiting lists).

The availability of tailored technical support was viewed as needed and important, and there was an appreciation for the support provided through eHealth colleagues. The newly implemented pathways, shortly after being launched, experienced technical issues from the service side that prevented patients from accessing links. Staff worked around this problem by sending new appointments and, ultimately, offering an in-person appointment. Eventually, the eHealth team resolved the problem’s source, but a few patients could not join despite trying multiple times. Initially, clinical leads also reported a problem with creating user accounts with the system using their NHS email addresses, which was promptly resolved. When at work, for improved efficiency, clinicians used one monitor for viewing digital appointments and another for the patient appointment booking system. However, this dual-monitor setup was not accessible when working from home:

So, when we’re trying to test something, we’ve got to get everyone involved in the test process, we have to get everyone to change everything at the same time, and then if the test doesn’t work then we need to go back up through the chain to find out whereabouts it failed.

Technical expert or lead 2

#### Outer Environmental Context

Staff believed that the pandemic created a favorable environment for telemedicine in general, a perspective that was in line with the experiences of the public. The pandemic acted as the catalyst for thinking about it on a larger scale. However, it instigated an increased demand and competition for resources. Participants mentioned the video consultation service used in Grampian called *Near Me*. Even before the pandemic, they said, it was viewed as an effective new method of teleconsultation, which paved the way for teleconsultation innovation and served as a benchmark with which adopters compared the asynchronous consultation system:

Although we had been looking at it prior to Covid because we thought this was a way of working anyway, but Covid was the catalyst that got us thinking about it on a larger scale.

Technical expert or lead 1

In the backdrop of dealing with Covid, it’s difficult in terms of the pressure within the system to try and deliver something which was new and maybe something that people were just trying to learn and implement.

Project management lead or expert 1

#### System Antecedents and Organizational Readiness

NHS Grampian was generally viewed as a context receptive to telemedicine and innovations, characterized by a recognized need to introduce a new care model and having technical expert or leadership and innovation high on its agenda. Staff can and do exchange knowledge internally and externally, which is conducive to innovating. However, the receptiveness of the environment for innovation would be further strengthened by a more clearly articulated strategic vision for priorities, more clearly defined roles and responsibilities of innovation and transformation teams, and streamlined processes (such as information governance):

I mean I think the main things I would say are that I think NHS Grampian has been very supportive of this.

Technical expert or lead 2

The reality is there’s a lot of things that are urgent and need to be done, so I think that it’s more from an innovative perspective creating a kind of culture of innovation and getting agreement that this is how we work for innovative things and understanding that process and really encouraging.

Project management lead or expert 2

At the time of implementation, pain management and gastroenterology systems were perceived as more likely to succeed than the dermatology system due to the belief that there were more staff supporters of the innovation in those clinics.

#### The Importance of Staff and Patients as Adopters of the New Service

The innovation was developed and implemented by motivated and keen innovative clinicians and facilitated by the staff’s ability to work across traditional professional and disciplinary boundaries, working around setbacks and task issues. Good uptake of the innovation by patients was viewed as critical to maintaining staff motivation:

The clinicians themselves are very motivated to changing how they operate and engage with new digital tools, so they’re highly motivated to do that and they know their domain very, very well.

Technical expert or lead 1

Efforts were made to select and target the right group of patients considering condition type, demographic characteristics, psychological factors, and lifestyle. They initially expected the system to work better for some patients (returning patients, working people, and those needing continuous low-level input) and less for others (those digitally excluded—no internet or email—and with preference or need for human contact). This belief persisted over time, although subsequently, the notion that the system functions best for medical conditions for which treatment is highly standardized arose:

[A clinical lead] felt was suitable for, as the first try at this and she also thought she’d have a high uptake because of the demographic, because of the age range and the working nature of the folk with the condition she felt that they were ones who were likely to not want to come into clinic.

Technical expert or lead 1

I do not see why in a properly selected group of patients or conditions it could not be adopted in other areas. Like, it would be really good for patients with endocrine problems because the majority of their management is pretty standardised.

Clinical lead 1

Patients’ understanding was considered critical to managing patients’ expectations and, subsequently, a good uptake. For example, patients were observed to refuse digital appointments when contacted ad hoc due to a lack of previous knowledge of the purpose of the call. The term “appointment” was viewed as a misnomer causing a lack of understanding among the patients, who viewed the term “appointment” as an arrangement to meet someone at a particular time and date. Concerns were also expressed that patients may rely only on information from an invitation letter (rather than an information booklet) or may not have understood all the details (eg, what information exchanged during the consultation is saved).

Staff quickly learned how to effectively communicate the new service to patients to improve their acceptance of it. At the time of conducting the interviews, administrative leads expressed their plans to further revise the information leaflets and update the external website. Patients’ preferences affected initial product specification through administrative staff’s experiences with patients’ preferences (indirectly) and exploring patient opinions earlier in the new pathways’ specification process (directly), although some staff members felt that greater patient input might have been beneficial:

We’ve done a lot of work in what the patient information leaflets were like, letters were like, when we’re phoning the patients how we’re explaining it to them. I think feedback from when patients did onboard, feedback on the reasons why they didn’t onboard. We took that into consideration and changed our information leaflets to change...simple things such as changing the wording.

Service or administrative lead 3

#### Assimilation by the System

The adult dermatology system went live in May 2020 relatively smoothly, attributable to the project being driven centrally as a part of the national pilot project, thus having more momentum and being ready to go live by the time the pandemic hit. A major issue was observed during the initial implementation phase when adopters noted and worked around an initially significantly high *did not attend* (DNA) rate. It was not until February to March 2022 that uptake rates increased, which was attributed to appointing an administrative operational support that “pushed” and monitored the system to ensure that patients identified for digital appointments had been offered them and were supported where required, improved clinical buy-in (with more dermatologists offering the digital appointments), and service management supporting and driving digital appointment use within the service:

I think they could all relate to this story of it being given a task and trying to implement it by a date and not hitting that date, and then feeling that you’re constantly trying to push something up a hill just to try and get it done. I don’t think this is necessarily exclusive to this particular bit of work; it just happened to be the focus of the evaluation for us.

Project management lead or expert 2

There were several setbacks to the implementation of the gastroenterology and pain management pathways, leading to delays in the system going live: first, a 4-month delay in procurement (attributed to suboptimal coordination of efforts between the innovation and transformation teams); then, a 6-month delay in technical integration caused by capacity issues (loss of technical personnel by the NHS Grampian eHealth team and availability of the external technical resource [National Services Scotland (NSS)] to undertake the development); and, finally, a 1-month delay due to technical issues (eg, information governance):

I think NHS Grampian didn’t act upon the emergency procurement process rapidly enough for us to get the window with NSS [National Service Scotland] when they had the capacity to do it, as I look back.

Technical expert or lead 1

It’s really been the bottleneck at NSS that’s caused a lot of delays, and then also some personnel changes at NSS which have resulted in the people who’ve got knowledge of how that integration works moving on to new positions.

Technical expert or lead 2

Over time, the reality of these setbacks caused staff to grow tired and frustrated, with some losing their confidence in this specific innovation. Contrary to expectations, the groundwork made through the adoption of the dermatology asynchronous consultation system was not easily transferable to the pain management and gastroenterology systems; despite involving the same developer and product, “the reality turned into that it was an absolute uphill battle to even get it live” (project management lead or expert 2):

I think the area that frustrated us more was the integration side of things because we already had integration up and running with dermatology and from our perspective it should’ve been a relatively simple process.

Technical expert or lead 2

Overall, almost all adopters described feeling surprised by the complexity and nonlinear nature of the process and implementation effort involved. Initially, this provoked reflections on the timing of technical integration, sequencing of steps, timing of stakeholder involvement, and lack of guidance on core and optional steps. After the implementation, this prompted recognition of the “need to have those champions and people that are pushing for it and really taking it through” (project management lead or expert 2):

If you integrate it too early, you might end up with something that won’t scale very well. If you integrate it too late, you might end up with lots of messy ways of working with lots of different workarounds that people don’t want to change. I think there is definitely a sweet point of when’s the right sweet spot to integrate and I don’t think we got that right with this one.

Technical expert or lead 1

#### Implementation and Routinization

While decision-making was devolved locally to pain management and gastroenterology clinical leads (whereas, in practice, dermatology was centrally led as part of a national pilot project), its adopters expressed regrets over missed opportunities to make implementation smoother by taking administrative staff’s concerns more seriously early on, especially in terms of system readiness and how to improve patient uptake:

Involvement of the stakeholders, as I say, the secretarial staff would have been absolutely brilliant at pointing out where the omissions might happen, or kind of documentation issues might happen that we would never foresee.

Clinical lead 4

The organization and delivery of the innovation were human resource intensive, requiring a high level of staff buy-in and administrative input. Allocated time was perceived as needed for clinicians, operational staff, clinic coordinators, project managers, evaluation leads, and eHealth teams. The appointment of administrative operational support was perceived as directly responsible for an increase in uptake. A need for continued funding and dedicated resources was stressed, but this was against extreme pressure on NHS resources generally:

If it was funded appropriately that would have maybe improved things as in funding for NHS staff and for NSS involvement. There was very little funding available within the NHS.

Technical expert or lead 2

There was a new appointment made a number of months ago, which has...actually, you can see it’s paid dividends now in terms of the take-up of the figures in terms of the digital appointments as well, but that’s kind of an admin support.

Project management lead or expert 1

Delays with technical integration prompted reflections on the role of the NSS integration hub as a national-level NHS stakeholder. The NSS appeared to have limited capacity for this project. The innovation was intended as a proof of concept rather than a fully operational system, so it needed more time commitment and a clear description of roles and contractual agreements on escalation routes. The delays in technical integration caused the technical leads to wonder whether an IT system at the proof-of-concept stage should be fully integrated with the host organization’s IT system immediately or only after it has undergone functional testing.

Incompatibilities with the current ways of working did not surface until after implementation, leaving adopters unsure of whether it “translates practically into the real world” (service or administrative lead 1). The main issue was the so-called “lifetime of an appointment” in the patient booking system. While in-person appointments are acted upon the same day of the appointment, asynchronous consultations would appear still booked, which was “kind of alien to [admin staff]” (service or administrative lead 2). Another issue concerned how clinicians plan their time, imposing limits on how flexibly the new system could be used (ie, managed by allocating time slots 2 afternoons a week or ad hoc but ultimately depending on patients’ timing). At the time the interviews were conducted, a solution for this issue had not been established:

I think the notion behind the asynchronous appointments or assessments, or the process is good. I don’t know that it translates practically into the real world if that makes sense. So, I think the idea behind it around you don’t need to have dedicated clinical sessions, you can spread these appointments out throughout the week and the clinicians can deal with them at any time, that doesn’t fit with the world we live in in terms of job planning and service planning and things.

Service or administrative lead 1

From early on, there was a perceived need for better 2-way communication between the project manager and adopters, including relevant people from the start (importantly, administrative staff) and keeping them informed throughout, keeping records of communication, and clearly outlining roles (knowing who to contact). Concerns about a perceived lack of a formal project board were aired—essential for acting on staff feedback brought up by a project manager. Transition or loss of personnel, especially project manager changes, significantly disturbed stakeholders’ involvement and communication continuity. Later, a new acting project manager addressed this by ensuring a 2-way communication line with the operational staff, which they felt helped restore trust.

#### Perceived Consequences

Initial low confidence in the success of the dermatology system improved with time following “quite a dramatic increase in Dermatology take-up in terms of the number of appointments that have gone through” (project management lead or expert 1).

As the new pathways went live, it was felt that some of the new pathways were better accepted by patients than others and that the new system would be more successful overall if it fit better with the existing process flow. For example, it was considered to work well for pain management because patients were scheduled in a sequence (when they reached the top of the list as opposed to being chosen by hand) and clinicians introduced patients to the service during their initial appointment:

Pain Management it works very well with them because they already used a similar system. The patients are being onboarded by the clinicians at the time, or the care providers at the time of their initial appointment so it’s been explained to them what it is and how it works.

Service or administrative lead 3

Overall, the new system was viewed as useful to many patients, but “there needs to be an alternative option because it’s not for everybody” (clinical lead 3). As for the implementation success, participants seemed to agree that “in the face of all the adversity and challenges that the work has faced, the fact that that still is getting used and patients are still buying into shows that, I guess, as an attempt to introduce this and upscale it, it can be done” (project management lead or expert 2)

### Assessment of Service Uptake by Patients

#### Assessment of Routinely Collected Data

The adult dermatology service was the first in NHS Grampian to offer digital asynchronous appointments to their patients in May 2020, followed by the pediatric dermatology service in January 2021. The gastroenterology and pain management departments started offering digital appointments in December 2021 (10 months later than originally planned).

The responses of patients offered digital appointments were based on an audit carried out by administrative staff between February and July 2022 (n=832). A total of 41% (341/832) of patients accepted and received digital appointments, only 16.9% (141/832) explicitly declined, and 31% (258/832) did not respond to the clinic letter offers or believed that they no longer required a consultation. The remaining 11.1% (92/832) comprised people whom services had already seen, people who had already booked a different type of consultation, and a small number of people classified as *other*.

There were a total of 1709 digital appointments offered to 1417 patients across the 4 medical specialties, between May 2020 and September 2022. The total outcomes (n=2416) were greater than the number of appointments (N=1709) because there could be more than one outcome per consultation. The overall mean age of service users (N=1709) was 38.7 (SD 22.7) years, ranging from 1 to 90 years. A total of 55.59% (950/1709) of patients were women, and 79.4% (1357/1709) were White. Moreover, 4.62% (79/1709) of people who chose to use this service were from our most deprived communities, whereas the figure in the Grampian region overall is 5.36% (31,433/586,530; Figure S1 in Multimedia Appendix 2). This discovery is important as both public and staff groups were concerned with assessing whether the system could potentially worsen health inequalities, a consideration that would be a major disadvantage.

DNA rates were high in the first 2 months that digital appointments were used in the pain management (7/21, 33%) and gastroenterology (17/38, 45%) services. On the other hand, once the service was established the overall DNA rate (between January and September 2022) was 13.7% (125/909), which is approximately 5% higher than the national face-to-face appointment DNA rates for gastroenterology and adult dermatology. There were apparent differences across medical specialties and time (Table 1), yet there was no apparent impact based on the length of time the specialty had been offering the new service. This would be evident if DNA rates in the dermatology pathways were generally lower.

**Table 1:** Did not attend rates across medical specialties using digital appointments in the first 3 quarters of 2022.

Medical specialties	Total appointments, n	January to March, n/N (%)	April to June, n/N (%)	July to September, n/N (%)
Gastroenterology	234	7/45 (15.6)	3/70 (4.3)	17/119 (14.3)
Pain management	83	3/16 (18.8)	4/23 (17.4)	8/44 (18.2)
Adult dermatology	385	16/132 (12.1)	24/131 (18.3)	23/122 (18.9)
Pediatric dermatology	207	5/36 (13.9)	13/135 (9.6)	2/36 (5.6)

Looking at the 4 medical specialties overall, there was no significant difference in DNA rates across the 5 SIMD deprivation quintiles (χ^2^_4_=7.8; *P*=.10). The actual DNA rates were as follows: 25.5% (79/310) for SIMD 1 (most deprived); 23.3% (82/352) for SIMD 2; 23.9% (78/327) for SIMD 3; 20.7% (78/377) for SIMD 4; and 17.3% (58/335) for SIMD 5 (least deprived). SIMD could not be calculated for 8 cases.

The most common outcome from appointments overall was open returns (ie, that a patient can request another appointment if and when they feel they require one; 1208/2416, 50%), followed by treatment (894/2416, 37%); discharged (242/2416, 10.02%); and, finally, referred on (72/2416, 2.98%). There was no evidence that deprivation levels impacted on whether people were offered treatment following their appointments, with proportions similar across quintiles (χ^2^_4_=2.7; *P*=.61). The numbers offered treatment were as follows: SMID 1 (177/894, 19.8%), SIMD 2 (166/894, 18.6%), SIMD 3 (182/894, 20.3%), SIMD 4 (193/894, 21.6%), and SIMD 5 (176/894 19.7%).

In terms of the estimated environmental impact, assuming people would travel from their home address (area spread across 3000 square miles) to where the Aberdeen Royal Infirmary is located, digital appointments resulted in 44,712 fewer miles (approximately 71,956.81 km) traveled than traditional face-to-face approaches.

#### Patient Experience

Of the 317 patients who attended digital appointments at the gastroenterology and pain management clinics, all of whom were offered to complete the web-based satisfaction survey, only 66 (20.8%) patients completed it. The vast majority of respondents (57/66, 86%) were patients attending the pain management service.

Approximately 1 in 5 of patients were unhappy or very unhappy to be offered a digital appointment (12/66, 19%) and a similar proportion continued to feel the same way about using this type of appointment after they had experienced it (14/66, 21%; Table 2).

**Table 2:** Results of the satisfaction survey with a digital appointment from those who completed their appointment (n=66).

Survey questions	Very happy, n (%)	Happy, n (%)	Neutral, n (%)	Unhappy, n (%)	Very unhappy, n (%)
How did you feel about being offered a digital appointment?	11 (17)	22 (33)	21 (32)	7 (11)	5 (8)
How do you feel about using digital appointments for this type of health care service now that you have tried it?	8 (12)	26 (39)	18 (27)	8 (12)	6 (9)

The vast majority (59/66, 89%) of patients believed to a large or very large extent it is important to be involved in decisions about their care; however, much fewer felt to a large or very large extent that they were involved in their digital appointments (23/66, 35%; Table 3). Two-thirds (44/66, 67%) rated the quality of their care as excellent or good.

**Table 3:** Results of the satisfaction survey with a digital appointment from those who completed their appointment (n=66).

Survey questions	To a very large extent, n (%)	To a large extent, n (%)	Moderately, n (%)	To some extent, n (%)	Not at all, n (%)
How important is it to you to be involved in decisions about your health care?	47 (71)	12 (18)	5 (8)	1 (2)	1 (2)
How involved did you feel in the outcome of your digital appointments?	6 (9)	17 (26)	19 (29)	10 (15)	14 (21)

A total of 10% (6/66) of the survey respondents agreed to follow-up interviews. Follow-up interviews suggested that people saw a role for it in the NHS, viewing it as an additional, optional method alongside other forms of consultation, consistent with public opinions. Key specific benefits experienced included the time saved on travel and improved information collection and sharing, which both the public and staff anticipated:

I think that there are huge amounts of benefits in having this kind of system, but somewhere in it you have to include the ability to have a face-to-face.

P3; male

It saves you having appointments with Tom, Dick, and Harry where you can have it with Harry, and he can pass the information on to Tom and Dick.

P5; female

Some felt apprehensive about the offered service, fearing that it might eventually replace human connections, and overall preferred in-person communication. This sentiment is in line with the distinct preference for this type of contact expressed by some public focus group participants. They believed that teleconsultations distanced patients and health care professionals, emphasizing the significance of being physically present in creating a trusting physician-patient connection. Losing meaning when writing or even suppressing their answers were also mentioned. However, they acknowledged that the written format offers advantages, such as an opportunity to review a response and writing in one’s own words in a free-text box:

You’re being offered primarily video sessions and telephone sessions from your surgery. There has been a barrier created between the medical profession and Joe punter [a user].

P3; male

I think if you’ve got to write things, yeah, that looks bad if I’m saying, “No, this isn’t what I want.”

P2; female

It’s like a survey, you can ask certain questions but there may not actually be a response available that matches what you want to say. There are benefits to it because you have time to think through...so if for example, it’s a free text box, you have the opportunity to construct what you’re saying and then review your response.

P2; female

Those who were happy to be offered the service, on the other hand, stated that rushed in-person consultations lack the human factor anyway and saw digital appointments as an opportunity to improve relationships because physicians have a better understanding of their needs. They liked having time to think about and reconsider their comments; they thought that they covered things quite well and described the questions set as straightforward, straight to the point, and more personal; and saw it as an unavoidable change:

I had time to sit down and actually read the question and properly kind of, not prepare answers, but kind of rethink what my answers would be. Honestly, I think that would probably better the relationship because the doctors have a better understanding of the answers for the questions that they gave.

P6; female

I think these people have got to get up-to-date with the times...if that’s how it’s going to be, you’re going to have to change with the times or get someone to help you to change with the times.

P4; male

A total of 4 types of influences on the ease of use of the new service were mentioned: difficulty receiving timely care, condition, ability, and technical issues. More specifically, some patients had previously had difficulty receiving timely care. For some, this resulted in a preference for in-person contact, whereas for others, it enhanced appreciation for having faster access:

You’ve got to jump through so many hoops...By the time you get to say something, you’ve become incoherent as well.

P2; female

I was really looking forward to it, and I believed it would be useful.

P1; female

In total, 25% (1/4) of the interviewed participants who completed their appointments did so without assistance and felt the direction provided (an email with instructions) to be extremely clear. The remaining 75% (3/4) of the participants expressed doubt in their ability to complete the appointment without assistance, so “to be sure that [they] knew what to do” (P2; female), they sought assistance from a family member, carer, or employee. Consistent with the public views, patients thought that those with limited digital literacy and certain cognitive impairments and those who did not speak English as their first language would be least likely to use this service.

Some people’s ability to go to in-person appointments, remain seated, and even respond to the questionnaire was reported to be affected by their condition. In line with the public’s perception of possible disadvantages, some people found it difficult to convey their health status, particularly pain, in words, necessitating physical contact with a clinician:

Because I’m in pain, I mean, I can’t sit in the same place. I’m afraid you just stop listening.

P1; female

When I filled in that, I could quite well be contradicting myself by what I’m saying now. You’re not well, things change.

P2; female

In total, 2 participants reported experiencing technical issues (the link to the appointment not working, poor broadband connection, and a “device that refused to work” [P3; male]). One participant thought that was normal and resulted from “a lack of experience on both sides” (P3; male). Another participant felt confused and “not being taken seriously” (P2; female). Those patients sought assistance from the clinic, which eventually provided options for in-person and telephone consultations. This approach is in line with public opinions on the necessity of providing alternative forms of consultation and aligns with staff reports on actions taken to sidestep technical issues.

Consistent with staff views, we noted that the primary area for improvement was participants’ comprehension of the service. All but 1 patient reported having trouble memorizing and naming specific phases in their treatment pathway, indicating difficulties with eHealth terminology and the complexity of the pathway. Patients did not consider the *digital appointment* as an appointment but rather as an exchange of information or assessment to determine “whether [they] actually need an appointment” (P2; female). Most respondents who completed the consultations did not recall receiving a consultation copy, which indicates that this information was not effectively communicated. In addition, there was no clear connection perceived between the outcome of the consultation and the responses made, indicating a lack of knowledge of how information obtained from consultations influenced treatment decisions.

## Discussion

### Principal Findings

A range of patients decided to use the asynchronous consultation service of the dermatology, gastroenterology, and pain management departments, with a total of 1709 appointments offered to 1417 patients from May 2020 to September 2022 across 4 medical specialties. The public was overall receptive to the service, and 82% (54/66) of real users felt neutral to very happy about being offered the service. Improved access, ease, information collection and sharing, and more physician flexibility were among the benefits highlighted. The main concerns expressed by both the public and staff was the fear of digital exclusion, potentially worsening health inequalities, and the loss of human contact. However, NHS data showed no evidence that people from the most deprived areas were less likely to accept digital appointments, receive treatment, or be given open return appointments. Regarding human contact, some patients preferred direct interaction, whereas others noted that face-to-face encounters did not always enhance connection and found written communication to be more effective for clinician understanding. Recognizing that it may not work for certain individuals, especially those with limited digital resources or writing abilities, and that other people would prefer in-person interaction, informed choice and flexibility are required. The administrative processes could be improved, starting with better communication to promote patient acceptability by changing the term *digital appointment* to *assessment*.

The adopters (staff) expected a relatively straightforward technical system transfer from dermatology to other receptive specialties, with generally positive support from the clinical and administrative staff tasked with implementing it. Despite a favorable context and organizational support, the reality proved immensely complex. Nonetheless, staff understanding of the asynchronous system, how to describe it to patients, and how to use it to the best effect evolved rapidly. They perceived variances in adoption among specialties, with pain management being regarded to function better (easier to use) as the new approach suited present practices, but numerical data did not show that the approach functioned any differently for pain management clinics. The sustainability of this new system was seen as linked to its transferability across many specialties and the availability of funds to afford allocated continuous time for clinicians, operational staff, clinic coordinators, project managers, evaluation leads, and eHealth teams.

### Limitations

There was a substantial unanticipated delay in implementing the system, which is a finding in itself. The NHS is a system under constant pressure with limited support for innovation available, so the risk of delays seems difficult to eliminate. We used this chance to capture and report on implementation challenges, and the NHS Grampian partner’s commitment to transparency in reporting the implementation process made this feasible. However, the implementation delay meant that there was less time than we had hoped to recruit patients to interview who had completed the appointment process, and as a result, our sample of patient interviews was smaller than we had planned. The number of people who were able to complete the NHS satisfaction survey was also small and limited to the gastroenterology and pain management clinics (due to permission restrictions). The NHS Grampian data have limitations as well—we were unable to collect any data on patient outcomes; however, our focus was on assessing the implementation approach across specialties rather than on specific patient outcomes within individual pathways.

### Comparison With Prior Work

In accordance with a global agenda [20], the UK Department of Health and Social Care [21] and Scotland’s Digital Health and Care Strategy [22] aspire to expand the range of digital clinical and care services and ensure that staff can work remotely and flexibly. Asynchronous patient-to-physician communication offers that. Previous studies have revealed that teleconsultations are generally acceptable to NHS patients [23]. We found that the public is receptive to NHS outpatient asynchronous consultations if individual preferences are respected, the system is suited to end users’ needs, and the service is optional. Most outpatient asynchronous consultations involve submitting a response to a set of key questions about a condition and uploading photos or videos (eg, for dermatology [9] or epileptic seizures [11]), but overall, asynchronous consultations have a variety of uses across countries [6]. An asynchronous consultation service for pain management and gastroenterology care involving a set of key questions only is a viable and acceptable option.

Consistent with what we know already from the wider implementation literature [18], future implementation efforts regarding digital innovations should consider assessing ahead of implementation key organization-wide factors identified by our staff participants as desirable, including a degree of consensus on broader innovation priorities and protocols, clearly defined roles and responsibilities of innovation and transformation teams, adequate resources and involvement of stakeholders, and strong and continuous project management. Further research is needed to help organizations build and implement evidence-informed strategies to prioritize and manage innovation.

The nonlinear and complex nature of the implementation process has been previously reported [18]. This study resonates with that and further suggests that it can be expected even when the same product is scaled up in the same setting. However, the reality of staff’s frustrations with the complexity of that process is less documented. It is widely recognized that scaling innovation takes space, time, and resources, so organizations need to consciously and strategically drive scaling efforts [24]. We noted the critical importance of numerous influences on clinicians’ motivation to adopt and continue engaging with the innovation (eg, uptake by patients, competing priorities and time pressures, exposure to the innovation work, and continuous 2-way communication with a project manager). In the primary care setting, the importance of compatibility with the existing workflow and patient satisfaction with asynchronous consultations was also noted [6]. It is crucial to include administrative staff issues early on as they may anticipate patient ease of use and compatibility with the existing workflow.

The importance of the involvement of end users is reported in the eHealth literature in general [25] and in chronic disease management [26] specifically, emphasizing the need for user-centered design. The importance of providing user-friendly, role-appropriate information and resources to support the individual being cared for [27] and patient involvement in digital service design from the outset have been noted [6,28]. This can be done with the help of one of many frameworks for gathering insights from patients and the public [29]. This study echoes this and emphasizes the need to understand and tackle digital exclusion and provide informed digital choice via system cospecification and effective communication regarding outpatient asynchronous consultations.

The current body of research points to a rising interest in patient safety within telemedicine, although there is a lack of detailed studies on this topic [30]. Consistent with this trend, this perspective warrants attention in this research. In this study, the team responsible for implementation assessed and monitored the new service, worked on their communication with patients, ensured data security and system resilience, and maintained follow-up protocols, which are all key elements of patient safety [31]. We reported issues concerning whether patients fully comprehend the nature and content of the communication process, which is vital for patient safety [31]. Overall, this study supports the relevance of establishing technical standards and guidelines to guarantee safety and quality in asynchronous consultations as a form of telemedicine.

### Conclusions

This research shows the viability and great potential of asynchronous consultations and wider digital solutions in the NHS to be a key part of meeting increased demands on the NHS. Recognizing that it may not work for everyone, flexibility and informed choice are key. For potential patients, careful technical support and explanation are needed, as well as a choice of consultation routes, to ensure digital inclusion. These findings also highlight how essential effective patient and public involvement is for the success of any digital technology developments in the NHS, especially as the use of digital technology in health continues to rapidly advance. Our results on implementation effort complexity, the delays that occurred, the characteristics of the innovation, and its reception by hospital staff may help staff deploy and sustain asynchronous consultations.

## Appendix

Multimedia Appendix 1 Study tools (topic guides for public focus groups, staff and patient interviews, along with the patient satisfaction survey).

Multimedia Appendix 2 Supplementary data.

## Abbreviations

DNA: did not attend
NHS: National Health Service
NSS: National Services Scotland
SIMD: Scottish Index of Multiple Deprivation

## Data Availability

All relevant data can be found within the paper and Multimedia Appendix 2.

## References

[1] Monaghesh E, Hajizadeh A (2020). The role of telehealth during COVID-19 outbreak: a systematic review based on current evidence. BMC Public Health.

[2] Bitar H, Alismail S (2021). The role of eHealth, telehealth, and telemedicine for chronic disease patients during COVID-19 pandemic: a rapid systematic review. Digit Health.

[3] Horton T, Hardie T, Mahadeva S, Warburton W (2021). Securing a positive health care technology legacy from COVID-19. The Health Foundation.

[4] Dixon RF (2010). Enhancing primary care through online communication. Health Aff (Millwood).

[5] Bishop TF, Press MJ, Mendelsohn JL, Casalino LP (2013). Electronic communication improves access, but barriers to its widespread adoption remain. Health Aff (Millwood).

[6] Fuster-Casanovas A, Vidal-Alaball J (2022). Asynchronous remote communication as a tool for care management in primary care: a rapid review of the literature. Int J Integr Care.

[7] Mold F, Hendy J, Lai YL, de Lusignan S (2019). Electronic consultation in primary care between providers and patients: systematic review. JMIR Med Inform.

[8] Wherton J, Greenhalgh T, Shaw SE (2021). Expanding video consultation services at pace and scale in Scotland during the COVID-19 pandemic: national mixed methods case study. J Med Internet Res.

[9] Jiang SW, Flynn MS, Kwock JT, Nicholas MW (2022). Store-and-forward images in teledermatology: narrative literature review. JMIR Dermatol.

[10] Wherton J, Greenhalgh T Evaluation of the Near Me video consulting service in Scotland during COVID-19, 2020. The Scottish Government.

[11] SHTG assessment. Scottish Health Technologies.

[12] Nguyen OT, Alishahi Tabriz A, Huo J, Hanna K, Shea CM, Turner K (2021). Impact of asynchronous electronic communication-based visits on clinical outcomes and health care delivery: systematic review. J Med Internet Res.

[13] Nittari G, Savva D, Tomassoni D, Tayebati SK, Amenta F (2022). Telemedicine in the COVID-19 era: a narrative review based on current evidence. Int J Environ Res Public Health.

[14] Atherton H, Ziebland S (2016). What do we need to consider when planning, implementing and researching the use of alternatives to face-to-face consultations in primary healthcare?. Digit Health.

[15] Turner A, Morris R, Rakhra D, Stevenson F, McDonagh L, Hamilton F, Atherton H, Farr M, Blake S, Banks J, Lasseter G, Ziebland S, Hyde E, Powell J, Horwood J (2022). Unintended consequences of online consultations: a qualitative study in UK primary care. Br J Gen Pract.

[16] Palinkas LA, Horwitz SM, Green CA, Wisdom JP, Duan N, Hoagwood K (2015). Purposeful sampling for qualitative data collection and analysis in mixed method implementation research. Adm Policy Ment Health.

[17] Chan S, Li L, Torous J, Gratzer D, Yellowlees PM (2018). Review of use of asynchronous technologies incorporated in mental health care. Curr Psychiatry Rep.

[18] Greenhalgh T, Robert G, Macfarlane F, Bate P, Kyriakidou O (2004). Diffusion of innovations in service organizations: systematic review and recommendations. Milbank Q.

[19] Population estimates by Scottish Index of Multiple Deprivation (SIMD). National Records of Scotland.

[20] Global strategy on digital health 2020-2025. World Health Organization.

[21] (2022). A plan for digital health and social care. Department of Health and Social Care, United Kingdom Government.

[22] (2021). Digital health and care strategy. Scottish Government.

[23] O'Cathail M, Sivanandan MA, Diver C, Patel P, Christian J (2020). The use of patient-facing teleconsultations in the national health service: scoping review. JMIR Med Inform.

[24] Albury D, Beresford T, Dew T, Horton T, Illingworth J, Langford K (2018). Against the odds: successfully scaling innovation in the NHS. The Health Foundation.

[25] Ahmed B, Dannhauser T, Philip N (2019). A systematic review of reviews to identify key research opportunities within the field of eHealth implementation. J Telemed Telecare.

[26] Taylor ML, Thomas EE, Vitangcol K, Marx W, Campbell KL, Caffery LJ, Haydon HM, Smith AC, Kelly JT (2022). Digital health experiences reported in chronic disease management: an umbrella review of qualitative studies. J Telemed Telecare.

[27] Enabling, connecting and empowering: care in the digital age. Scottish Government and COSLA.

[28] Baines R, Bradwell H, Edwards K, Stevens S, Prime S, Tredinnick-Rowe J, Sibley M, Chatterjee A (2022). Meaningful patient and public involvement in digital health innovation, implementation and evaluation: a systematic review. Health Expect.

[29] Bird M, McGillion M, Chambers EM, Dix J, Fajardo CJ, Gilmour M, Levesque K, Lim A, Mierdel S, Ouellette C, Polanski AN, Reaume SV, Whitmore C, Carter N (2021). A generative co-design framework for healthcare innovation: development and application of an end-user engagement framework. Res Involv Engagem.

[30] Guise V, Anderson J, Wiig S (2014). Patient safety risks associated with telecare: a systematic review and narrative synthesis of the literature. BMC Health Serv Res.

[31] de Micco F, Fineschi V, Banfi G, Frati P, Oliva A, Travaini GV, Picozzi M, Curcio G, Pecchia L, Petitti T, Alloni R, Rosati E, De Benedictis A, Tambone V (2022). From COVID-19 pandemic to patient safety: a new "spring" for telemedicine or a boomerang effect?. Front Med (Lausanne).

