# Supplementary material for: Implementation of a Web-Based Outpatient Asynchronous Consultation Service: Mixed Methods Study
Source: J Med Internet Res. 2024 Jun 4;26:e48092. doi: 10.2196/48092 (PMC11185905; doi:10.2196/48092)
Supplement: Multimedia Appendix 1 [file jmir_v26i1e48092_app1.pdf]

This is a **Multimedia Appendix 1** to a full manuscript published in the J Med Internet Res.

Study Tools

A) Public focus group semi-structured topic guide

We will start with a short description/presentation of an 'asynchronous' consultation followed by an invitation for participants to talk about their initial thoughts/feelings about it, so that we capture what matters to them in their own words.

Further prompts are likely to include questions like:

- How would you feel about using this type of consultation for health care?
- Are you aware of any technologies similar to this in healthcare and are any of you using them?
- What do you think might be the benefits of this form of consultation, for you or other people? What might the disadvantages be?
- How could this kind of consultation be improved?
- What barriers are there for wider use of technology like this in the NHS?
- What can be done to overcome these barriers? Who needs to take actions (e.g., government, developers, NHS, user groups)?

B) Staff interview questions

Opening question:

- "Tell me about your experience using asynchronous consultations"

Indicative semi-structured prompts regarding the pre-implementation stage:

- What is new about this system?
- How are you working with other colleagues to implement the system?
- What added value do you think a system like this may bring if any?
- How are you monitoring its impact?
- Who 'asynchronous' consultations might work for, and when?
- How compatible 'asynchronous' consultation is with your current workflow?
- What resources are required to implement this form of consultation?

Indicative semi-structured prompts regarding the post-implementation stage:

- What's worked well?
- What's worked less well and why?
- How have patients responded to using it?
- How well has it been possible for you to develop or maintain a relationship with your patients using the system?
- How has the team as a whole felt about the system?
- What role do you think the system will play as part of the board's offering to patients in future?
- How has it changed over time and what are its effects?
- What are the benefits and drawbacks you can see for staff and for patients?

Indicative semi-structured prompts at both stages:

- How did the initial expectations compare with reality?
- What were the key barriers that needed to be overcome and how for this to be integrated into routine practice?
- What modifications in practice and the system are required to make it sustainable?

C) Patient interview questions

A conversational style of interviewing will be adopted, to encourage a comfortable and fluent dialogue which is rich in detail, while using a semi-structured interview topic guide as a reference to ensure that all key topics were covered. Consistent with good qualitative research practice, prompts will evolve based on earlier interviews and depending on how the conversation with each individual is going.

Interviews will start with an open invitation to participants to "tell me about your experience of using the_'asynchronous' consultation process", unprompted by the researcher, so that we capture what matters to them in their own words.

Further semi-structured prompts are likely to include:

- What were your reactions when you were first invited to use the system?
- What were you expecting before you started using the system? How did this compare with the reality?
- How easy did you find using the system?
- Were there any technical or equipment issues which made it difficult?
- Did you involve friends and family to help you?
- How do you feel it affected the relationship with your doctor?
- What did you think of how he or she communicated with you when they gave you their response?
- Do you have suggestions for how the system could be improved?
- How would you feel about using it again?

As noted above, these prompts are indicative only and will vary in each interview, dependent on the participant's experience and their opening narrative.

D) Patient Satisfaction Survey (Declined)

Digital Appointment Patient Feedback Question Set 1

We try to make our services as helpful, timely and accessible as we possibly can. An important part of doing that is to ask people like you about their views. We would be extremely grateful if you would complete this short feedback questionnaire, to help us

provide the best possible service to our community. It should take only about a minute or so.

Required

1.Please let us know your forename e.g. Joanne

2.Please let us know your surname e.g. Smith

3.Please let us know your date of birth

4.Please confirm your postcode e.g. AB21 8HL

5.Can you please indicate with which service you were offered a digital appointment by clicking on the box beside the options

- Gastroenterology
- Pain Management Service

6.Can you please tell us why you decided to not use our digital appointment system by clicking the box beside the options. If you select "Other" then you can type your reason into the box provided. (Please note you can select multiple answers)

- Lack of privacy
- Poor connection to the internet
- Lack of time to complete the questionnaire
- Everything seemed complicated
- I don’t like using technology for this type of thing or in general
- I want to speak to a real person face to face

7.Would you be happy to discuss your thoughts about digital appointments further with a researcher (Magda Rzewuska. Email: magdalena.rzewuska@abdn.ac.uk) from the University of Aberdeen? This conversation would be with someone outside your NHS care team, remain confidential, and would not impact on your care in any way. The researcher would contact you directly.

- No
- Yes

Submit

E) Patient Satisfaction Survey (Did Not Attend)

Digital Appointment Question Set 2

We try to make our services as helpful, timely and accessible as we possibly can. An important part of doing that is to ask people like you about their views. We would be extremely grateful if you would complete this short feedback questionnaire, to help us provide the best possible service to our community. It should take only about a minute or so.

Required

1.Please let us know your forename e.g. Joanne

[ ]

2.Please let us know your surname e.g. Smith

[ ]

3.Please let us know your date of birth

[ ] ?

4.Please confirm your postcode e.g. AB21 8HL

[ ]

5.Can you please indicate with which service you were offered a digital appointment by clicking on the box beside the options

- ○ Gastroenterology
- ○ Pain Management Service

6.Can you please tell us why you decided to not use our digital appointment system after opting in to this service by clicking the box beside the options. If you select "Other" then you can type your reason into the box provided. (Please note you can select multiple answers)

- ☐ Lack of privacy
- ☐ Poor connection to internet
- ☐ Lack of time to complete questionnaire
- ☐ Questions didn't make sense
- ☐ Questions didn't seem relevant to my situation
- ☐ Everything seemed complicated
- ☐ I don't like using technology for this type of thing or in general
- ☐ I want to speak to a real person face to face
- ☐ I forgot about the appointment
- ☐

[ ]

7.Would you be happy to discuss your thoughts about digital appointments further with a researcher (Magda Rzewuska. Email: magdalena.rzewuska@abdn.ac.uk) from the University of Aberdeen? This conversation would be with someone outside your NHS care team, remain

confidential, and would not impact on your care in any way. The researcher would contact you directly. Please click the box beside the options

- No
- Yes

Submit

F) Patient Satisfaction Survey (Completed appointment)

Digital Appointment Question Set 3

The survey will take approximately 4 minutes to complete. We try to make our services as helpful, timely and accessible as we possibly can. An important part of doing that is to ask people like you about their views. We would be extremely grateful if you would complete this short feedback questionnaire, to help us provide the best possible service to our community. It should take only about a minute or so.

Required

1.Please let us know your forename e.g. Joanne

2.Please let us know your surname e.g. Smith

3.Please confirm your date of birth

4.Please confirm your postcode e.g. AB21 8HL

5.Can you please indicate with which service you were offered a digital appointment by clicking on the box beside the options

- Gastroenterology
- Pain Management Service

6.How did you feel about being offered a digital appointment?

- Very Unhappy
- Unhappy
- Neutral
- Happy
- Very Happy

7.How do you feel about using digital appointments for this type of health care service now that you have tried it?

- Very Unhappy
- Unhappy

- Neutral
- Happy
- Very Happy

8.How involved did you feel in the outcome of your digital appointments

- Not all all
- To some extent
- Moderately
- To a Large Extent
- To a Very Large Extent

9.How important is to you to be involved in decisions about your health care?

- Not at all
- To Some Extent
- Moderately
- To a Large Extent
- To a Very Large Extent

10.Overall, how would you rate the quality of the healthcare you received

- Very Poor
- Poor
- Fair
- Good
- Excellent

11.Is there anything else you would like us to know (free text)

[ ]

12.Would you be happy to discuss your thoughts about digital appointments further with a researcher (Magda Rzewuska. Email: magdalena.rzewuska@abdn.ac.uk) from the University of Aberdeen? This conversation would be with someone outside your NHS care team, remain confidential, and would not impact on your care in any way. The researcher would contact you directly. Please click the box beside the options

- No
- Yes

Submit
