# Supplementary material for: Implementation of a Web-Based Outpatient Asynchronous Consultation Service: Mixed Methods Study
Source: J Med Internet Res. 2024 Jun 4;26:e48092. doi: 10.2196/48092 (PMC11185905; doi:10.2196/48092)

This is a **Multimedia Appendix 2** to a full manuscript published in the J Med Internet Res.

Supplementary data

Table S1. Characteristics of participating patient interviewees.

ID	Stage	Pathway	The sentiment expressed in the survey	Self-reported characteristics (gender; age; ethnicity; rurality; employment status; living alone/not; satisfaction with digital appointment)
P1	did not attend	pain self-management	very unhappy (was looking forward to it but had technical issues)	female; age group 55-64; white-British; urban (AB25); unemployed; living with a spouse; not so satisfied
P2	completed	pain - Qutenza	very unhappy (prefers in person)	female; age group ≥65; white-British; rural (AB56); retired; living with a spouse; not so satisfied
P3	did not attend	pain self-management	very unhappy (technical issue and prefers in person)	male; age group ≥65; white-British; urban (AB10); retired; living with spouse & children/grandchildren; somewhat satisfied
P4	completed	pain self-management	happy	male; aged group 55-64; white-British rural (AB53); working - business owner living with spouse & children/ grandchildren; not so satisfied
P5	completed	pain self-management	happy	female, age group 55-64; white-British; urban (AB16); retired; living with a carer; somewhat satisfied
P6	completed	pain self-management	very happy	female; age group 18-24; white-British; rural (AB31); working – business owner; living with a spouse; extremely satisfied

Box S1. Summary of thematic analysis of public perspective on asynchronous consultations.

<u>COVID-19's impact on the digital revolution</u>

"I personally gained a benefit when I wanted to see a GP and the receptionist said, "Oh, do you know we've got this web consultation process? Go the website", and I've got the desktop here, I've also got an iPad, clicked the button and within 15 minutes I was speaking to a GP, and that was something new and that was good." (FG1, #4 male)

"I think for me particularly over the last year for people who have suffered from isolation it's been a really good thing because it has allowed people to continue to communicate and not be so isolated. For me personally it's been very good"(FG2, #2 male)

- The COVID19 pandemic accelerated the uptake of digital technology, in this case telemedicine (especially synchronous consultations), enabling the digital transformation of the NHS
- Some people welcome the spread of digital technology; others fear the impact on the fabric of society ('addictive', 'intrusive', 'the social interaction side of things')
- Some participants personally experienced benefits (such as convenience) and downsides (such as it being hard to use for people with autism) from remote forms of consultation (such as the Near Me system for online video appointments in Scotland)

<u>Attitude towards asynchronous consultations</u>

"But it's good, it's great, I think it's a good idea and it would benefit a lot of folk, especially folk out of town as well like you said, Peterhead or up and down the coast and that." (FG1, #5 male)

"I had a lady just last week who went on to try and do the universal credit claim, but she doesn't want a phone, she doesn't want a computer, I mean she's 54/55, she doesn't want it, she's dead set against it, and I think there's a lot of people like her, it's not part of their life, they don't want to learn anymore, they've had enough, so I think the barrier's right there." (FG2, #5 female)

"I think even with ongoing conditions though I would be concerned if I was always getting a… I think there needs to be well once every six months or whatever you'll see a face to face because I have a skin condition that can flare up and I get lumps under my armpits, but that's… how do I know that that is just still a lump and it's nothing more?" (FG4, #3 female)

- Most participants were very enthusiastic about this new service and saw a place for it in the NHS (e.g. for long-term conditions but including an in-person follow-up), outlooks ranged from 'enthusiastic supporters' to 'cautious optimists'
- They predicted that some patients might choose to opt-out of the service due to fear/dislike of new technologies

<u>Relative advantages and disadvantages of asynchronous consultations</u>

"I have a lady who goes… she's got a problem with her feet in the [name of the charity], she has to go to [street name] every couple of weeks from [city's suburban area] and a taxi costs her £30 each time, she can't recoup that, that's her disability living allowance taken up with these appointments, and unfortunately she isn't online." (FG1, #1 male)

"Yeah, if it's cutting down on waiting times, or, then surely then the people who want to see people face to face whether it's culturally or generationally or just because there are barriers

to their accessibility choosing, then surely they would then get their face to face appointment quicker because there is less people going for face to face appointments." (FG2, #4 female)

"So I think sympathy, empathy but also human conversation, we're going to… these are all things that we have to work on, we work empathy, we work on sympathy, it's about taking that pause and the time, whereas a computer doesn't know to do that, allowing somebody to actually communicate. It's different putting down in one sentence or you've got 24… well 240 cases, whatever it is, see I don't even know this internet jargon, but I mean how can you put your condition in three sentences? That's what I worry about, I worry about the why and the reasons behind it I guess." (FG2, #6 female)

"Well also, sorry, digital inclusion is a massive… digital poverty is a massive issue for people where a lot of people would be willing to but they don't have the digital equipment to do that" (FG4, #3 female)

- Possibly shorter waiting time
- Fewer people on a waiting list for other forms of consultation
- Easier access for people living remotely and physically unwell/unable to travel
- Less hassle (less patient travel, no need to take a day off from work) and as a result, patients saving money and services have lower non-attendance rates
- More flexibility of time for staff and patients
- Very useful for people anxious or unable to express themselves in face to face or phone contact
- Less useful for people who don't speak English (proposed solution: translation service), with visual impairment and learning disabilities/ developmental disorders, chaotic lifestyles
- Participants reflected on the potential impact on quality of communication, including advantages (such as a patient having written notes improve clarity and are empowering) and disadvantages (such as conveying hard to explain problems, breaking bad news, lack of human interaction, care providers missing on practice in communication skills (proposed solution: voice activation function and telephone/video call option))
- Concerns about the risk of urgent cases missed (suggested solution: clear instructions needed, possibility to update information) and a threat to the holistic approach (proposed solution: integrated appointments in the future)
- People at risk of digital exclusion and their representatives struggled to think about asynchronous consultation as an appointment (they found the term 'web-based assessment system' better describes what the system does)

<u>Potential practical barriers and facilitators to using the asynchronous service</u>

"Well I just think if you're not computer literate and it's not a world that you're in that the whole process could cause that anxiety". (FG1, #5 female)

"Just that it's there and there's assurances that it's going to be all secure, but just a bit of fears around there has been some data breaches and I think that could be something that might put people off, but also then be something that they might be encouraged to use because it's less embarrassing in that sense, so it's kind of two ways. But also around the quality of the images, so I've got a smartphone but some of my family members, particularly my elderly relatives, they have a camera but it's not a really high quality image." (FG3, #4 female)

"So say they have got an asynchronous appointment and they're trying to fill in the information, in the booklet it says If you don't get an email or SMS to call the Aberdeen or Elgin team, but I'm just wondering about capacity for those that maybe have some technical issues and need that support" (FG3, #4 female)

- Technical barriers to use, such as no devices or electricity to charge them and no internet (proposed solution: government financial initiatives, third sector involved in patients transport, creating adequate spaces in pharmacies/GP practices)
- Lack of digital skills or insufficient skills (e.g., uploading photo on a computer)
- Other major technical issues: data protection and confidentiality, securing the data from damage, loss and unauthorised users
- Minor technical issues: system compatibility and data storage space required
- Persons' inability to use remote consultations should be recorded in GP electronic medical records to avoid having to pass multiple times through eligibility screening for use
- Reassuring system users that they can choose face to face or telephone contact at any time

Compatibility with needs

"I was thinking just about the example you've got on dermatology, how much other stuff could you go into? Would it just be something like that on the surface or any other condition? […] I think it would but more maybe rheumatology or something like that, an outward part of the body rather than inward with maybe a person how they walk, they stand, that type of thing." (FG1, #5 male)

"If we can offer a selection of ways in which people can access things, taking away the policies, the politics and the funding around internet access and exclusion in that way, if we can offer several different routes for people to be seen and heard and treated for non-urgent cases, then I can only see that as being a good thing (FG2, #4 female)

- May be unsuitable altogether if a condition needs physical examination, needs to be seen or people with 'chaotic lifestyles' (such as people with substance use problems)
- For some conditions, such as care of the elderly with complex needs, there were both advantages (e.g., less travel) and disadvantages (e.g., cognitive impairments, low digital literacy)
- It's a good option, but patients' preferences should be respected by the service remaining optional. In turn, some participants questioned how long it can remain an option and argued that the digital revolution is already here, and that people always feared technological advancements (such as electricity) like this but will eventually embrace them)
- Health and care equity mattered to participants - caution is needed to avoid lacking inclusivity or exacerbating existing inequalities
- Concerns about the fit with wider eHealth priorities

Table S2. A summary of determinants of the deployment of the asynchronous communication systems.

Determinants covered in our interviews	Determinants' status	Details uncovered through interviews	Illustrative quote
**CHARACTERISTICS OF THE INNOVATION ITSELF**			
**Relative advantage**	Potentially present	All interviewees reported potential beneficial consequences of the innovation: improved flexibility for clinicians and convenience for patients and enable timely access to the right care, a smoother system of information flow, informed resource allocation planning and environmental benefits<br><br>However, some potential disadvantages included: more workload; adding an unnecessary step; causing digital exclusion, loss of human touch	"I guess a sort of smoother system in that when the patients return their information, they'd be booked on to it." (#3 clinical lead)<br><br>"Patients are able to upload their information as and when, as opposed to coming in, travelling in, having a set time, they've got five days to upload this information." (#3 service/admin lead)<br><br>"Initially it was quite a lot of paperwork associated with it, so initially it probably wasn't all that much quicker than the phone call, but I would say now it is quicker than the phone call." (#3 clinical lead)<br><br>"The asynchronous appointments are very flexible for both the patient and the clinician, that's the whole idea" (#2 clinical lead)<br><br>"You know, we've got a better understanding of the impact of pain and their mood and their function, and how much it's interrupting daily activities, and that's not information that we would have had before we would have had to gained all the information during the first appointment." (#3 clinical lead)<br><br>"The first challenged I faced was that I wasn't getting any patients through because they were joining the routine waiting list [..] I had to really discuss this with management [..] now I vet them and they'll be offered a digital appointment the day that I vet it, so they don't have to wait 56 weeks for an appointment. If they take it, they will go digital. If they decline the digital appointment, they will join the waiting list for a normal appointment." (#1 clinical lead)

			"But this allowed me to slot them in, in a 15-minute appointment, instead of a 30-minute appointment, and my return list, which is much shorter in width in time than my new appointments, because I maybe would arrange for an investigation, so we have a digital appointment and see them" (#1 clinical lead)<br><br>"I think it does take away the personal aspect to seeing a clinician." (#1 service/admin lead)<br><br>"[in pain pathways] Patients are already engaged following a face-to-face appointment with the nursing teams prior to the async appointments. This provides a human touch and reassurance that patients' issues are known and will be considered, listened to." (#1 project management lead/expert)<br><br>"Also, the other things that have worked very well in my perspective is the fact that I have quite close interaction with them through the instant messaging system, and then I provide them a lot of information and outcome." (#1 clinical lead)
**Compatibility**	Potentially present	Pain and gastroenterology use asynchronous consultations for triage<br><br>Dermatology was viewed as driven by central rather than local needs	"So we have a team of psychologists and a physiotherapists now and we have developed a new flow into the service which includes an early information session about chronic pain and what the pain service offers and information on self-management. And, then, following this session, we screen patients using a questionnaire-based format that currently is delivered by a telephone consultation and then they are triaged to the most appropriate specialist to assess them, and we are using asynchronous in place of this telephone call." (#3 clinic lead)<br><br>"So the idea on the asynchronous clinic would be to try and triage these patients with a questionnaire trying to highlight what their main concern and symptoms are and then provide them with feedback of education, information, self-managed intervention and potentially in some patients write a letter to the GP and advise on medications." (#1 clinical lead)<br><br>"We want to kind of pop out that fairly early, but it's not really a clinic appointment in terms of our national returns and things like that, so that was

			that difficulty with it being so tied to the appointment meant that we didn't use it as widely as we could have done” (#1 technical expert/lead)<br><br>"Going back to the initial one with dermatology, this was originally a pilot within think, with hindsight, dermatology may not have been the best department to look at this application because I think they didn't have the same issues as Greater Glasgow and Clyde's dermatology department." (#3 technical expert/lead)
**Complexity – low degree of**	Partially present	The asynchronous journey process is perceived as disjointed for patients and some questionnaires are more complex than the others<br><br>Although early system-related technical issues that prevented patients from accessing connections were finally resolved, some patients are still unable to join (for technical reasons) despite numerous attempts	"My colleague was doing something that was a lot more complex, so lots of different arms to these questionnaires, whereas mine was kind of much more straightforward." (#4 clinical lead)<br><br>“It’s all about the patient and it’s about their experience, their journey. That’s where the focus needs to be, and it should come across very slick and very straightforward for them and very easy to use, because it’s just about the level of care that then is being provided to them and there shouldn’t be anything that stops that in that process or discourages them to use the process.” (#1 project management lead/expert)<br><br>“I guess, you know, initially some patients were having a problem accessing the link. The email that they were being sent out was a little bit confusing because they were clicking on the wrong link, so that meant a lot of patients hadn’t attended, so we said we would phone the patients that didn’t attend to check what the problem was, and if we couldn’t get through to them, we would write a discharge letter, we’d notify the digital team, so there’s less of those patients to follow up because the system is working better.“(#2 clinical lead)
**Trialability**	Absent	There was a perceived need to be able to try out the system before becomes a fully operational system	"I needed to go on and actually use the software, but we can't because we can't get logged into it." (#4 clinical lead)<br><br>“They can’t fully test things in Sky Store; they can only really test it when it eventually goes live, and that’s come up with specific issues like PDF issues that have arisen”. (#1 project management lead/expert)
**Observability**	Present	Pain and gastroenterology adopters reported benefiting from practical	"I guess just seeing [in the dermatology system] how we would organise a clinic for this digital consultation. I guess, how it presents to you, how do the

		demonstrations of the dermatology system, on how information is gathered, and patients are booked. Seeing different clinical case scenarios would have been helpful too<br><br>Formal training on how to use the system was offered to an asynchronous coordinator and clinical leads	patients…because it seemed very abstract as to how, we would know that patients had completed questionnaires" (#4 clinical lead)<br><br>"The asynchronous coordinator and our team leaders who have access as well, training for them to use the system. Our asynchronous coordinator's just recently been given access to both, the consultant side and the patient side of the system so they can see... have an insight, because if they're explaining to the patient what it is, they need to know what it is and how it looks so they can talk the patient through that. Not me, personally, because I haven't used the system, and I haven't got the access to do it, because that sits with the coordinator, but, yes, she will be showing me. I just had a chat with her" (#3 service/admin lead)
**Potential for reinvention**	Present	All adopters described the reinvention potential of the asynchronous consultation systems, in terms of target health condition and information formats, but technical restrictions to collected data were present	"Images, they're not required in this clinic." (#1 clinic lead)<br><br>"People have said they have concerns around their medication, so some of the questions are, "I'm concerned about the side effects." Or, "I'm concerned about the combination or the amount that I'm taking or the lack of effect." Then we look at their responses versus what their medication regime is, and if we're seeing, well, actually there's scope to make a change to this and we understand why they might have concerns [..] we're waiting to hear the evaluation of that but it might be that we want to add in a question that says, "Would you like to have an appointment with a medication review clinic to address your medication?" I think the information from the evaluation of that service will allow us to understand if we need to modify the questions a little bit." (#3 clinical lead)<br><br>"I use a lot of the messaging system. I think I'm the only one that uses it as much amongst the other users. But it's really important for me because these patients often have got complex symptoms, so I have to really quite try to pin down what I'm dealing with, and I also want to make sure that when I give them an outcome it's personalised to their needs." (#1 clinic lead)
**Task issues - low degree of**	Partially present	Clinical and admin staff reported time-consuming	"We ended up with like a spreadsheet, which is to me, stepping back in time in the way you operate, in order to phone patients, describe the process, try,

		and laborious extra tasks such as manual screening patients for eligibility and booking patients in for appointments (making phone calls and manual records keeping), more so for dermatology but as they became more familiar site some of the task became easier	and persuade them, get it booked and so on, and so it was very, very time consuming and clunky for a very, very small wedge of patients in comparison to the whole work of that area." (#2 service/admin lead)<br><br>"Ideally we would do the preparation first and it feels like we've gone back to front with it slightly and we're forever chasing our tail and getting patients into the clinics so the consultants can use the system." (#3 service/admin lead)<br><br>"We're just much slicker with that because we know what we're doing. I think it's just familiarising ourself with the system." (#2 clinic lead)<br><br>"Technical issues there haven't been that many in the grand scheme of things" (#3 service/admin lead)
**Technical support - presence of**	Present	Technical support was reported as available from a developer and eHealth Department	"So when we're trying to test something we've got to get everyone involved in the test process, we have to get everyone to change everything at the same time, and then if the test doesn't work then we need to go back up through the chain to find out whereabouts it failed." (#2 technical expert/lead)<br><br>"There's been a lot of support provided through eHealth colleagues." (#1 project management lead/expert)
**OUTER CONTEXT**			
**Socio-political climate - favourable**	Partially present	The pandemic accelerated uptake of remote consultations that paved the way for this system but also presented conflicting higher-order priorities and increased demand on staff	"Although we had been looking at it prior to Covid because we thought this was a way of working anyway, but Covid was the catalyst that got us thinking about it on a larger scale" (#1 technical expert/lead)<br><br>"In the backdrop of dealing with Covid, it's difficult in terms of the pressure within the system to try and deliver something which was new and maybe something that people were just trying to learn and implement" (#1 project management lead/expert)
**Incentives and mandates**	Absent	Healthcare innovation is not viable for small and medium enterprises that develop systems like this	"We make money from other sectors; we don't make any money in health [...] and the amount of time and effort that has to go into making these work is something that's just not viable for a lot of companies who've got a good idea or a good product" #2 technical expert/lead

SYSTEM ANTECEDENTS AND ORGANISATIONAL READINESS			
**Absorptive capacity for knowledge**	Partially present	In contrast to dermatology, pain and gastroenterology clinicians reported prior experience and knowledge of remote consultations. They also reported regularly discussing innovations at staff meetings and staff is a part of broader knowledge-sharing networks of colleagues with similar interests.	"Well the only consultation we use remotely is video links or telephone. We used video link like Near Me consultation for our patients much longer before the pandemic hit, so as an NHS board, NHS Grampian has always been ahead of everybody else" (#1 clinical lead)<br><br>"We didn't do many [remote consultations] prior to the pandemic. Near Me had just been created before it, but we weren't really using it regularly. Mainly, at the start of the pandemic, we switched to Near Me and telephone and then this pilot just started in the May time." (#2 clinical lead)
**Receptive context for change**	Partially present	NHSG is univocally viewed as supportive of innovations (e.g., for geographical reasons), with receptive digital leads and clinics keen to experiment with innovation<br><br>However, NHSG's receptiveness would benefit from a system-wide approach: a clear strategic vision for micro/macro/meso levels priorities, clearly described roles and responsibilities (e.g., between innovation and transformation teams); streamlined information governance and procurement processes;	"I mean I think the main things I would say are that I think NHS Grampian has been very supportive of this." (#2 technical expert/lead)<br><br>"Readiness for delivering it, I think in my opinion, it really hasn't been coordinated correctly. There are other initiatives which are being looked into for asynchronous appointments. There's was a Storm ID work in conjunction with the DHI, Digital Health Institute" (#3 technical expert/lead)<br><br>"And then the last bit in the mix there about receptiveness and readiness is around about information governance, so the information governance is still not signed off, unless it's happened in the last week. And there is something to learn about the processes about how we balance risk, so how do we balance those data risks versus the risks to the people who are out there that we're not seeing" (#1 technical expert/lead)<br><br>"The reality is there's a lot of things that are urgent and need to be done, so I think that it's more from an innovative perspective creating a kind of culture of innovation and getting agreement that this is how we work for innovative things and understanding that process and really encouraging" (#2 project management lead/expert)<br><br>"Clear role and responsibilities of innovation and transformation teams [would be helpful]" (#3 technical expert/lead)

		protocol (agreement that this is how we work for innovative thing), how to modernise an innovation once it becomes operational	
**Tension for change**	Present	Tension to reshape care model to reduce waiting lists and enable access to care for those in need	"It all started because our waiting list was really long and we needed to look at our referral over a year before but through COVID, and that's how this group started to work together" (#1 clinical lead)
**Innovation-system fit**	Partially present	The innovation does not fit well with the appointment booking system and clinician's use of time<br><br>Questions used by some of the pathways are aligned with the national agenda	"I think the notion behind the asynchronous appointments or assessments, or the process is good. I don't know that it translates practically into the real world if that makes sense. So I think the idea behind it around you don't need to have dedicated clinical sessions, you can spread these appointments out throughout the week and the clinicians can deal with them at any time, that doesn't fit with the world we live in in terms of job planning and service planning and things." (#1 service/admin lead)<br><br>"We've got a five-day period where an appointment is sitting at still booked on our system and that is kind of alien to us because that shouldn't be happening [..] It has caused us more administrative work [..] we have targets that we have to meet for that piece of work to be done, so my management team will run reports and we've had to sort of exclude all these Async appointments because ordinarily we would be chasing up appointments that are sitting as still booked on Track" (#2 service/admin lead)<br><br>"You might go in to look the day before and say, "I'll complete those now because I've got a bit of spare time." But actually, the patient might not have submitted their responses." (#3 clinical lead)<br><br>"Pain Management it works very well with them because they already used a similar system. The patients are being onboarded by the clinicians at the time, or the care providers at the time of their initial appointment so it's been explained to them what it is and how it works. [..] For Dermatology and Gastro, those other two specialties, we have a backlog so patients aren't being seen when they're supposed to be, so we're booking people out of order and so we're able to fill the clinics so we can see how the process works and for

			the consultants to use the system to get used to it, we're booking people out of order and we don't have alternative appointments to offer the". (#3 service/admin lead)
**Power balances - more supporters than opponents**	Present	Reportedly there were more supporters than opponents and innovation champions in leading positions in pain and gastroenterology (but not dermatology)	"It was really the clinicians that I was asked just to make sure that we had them onboard, so for my own specialty I was doing the coordination work" (#1 technical expert/lead)<br><br>"Dermatology was one key consultant, and in my view it suited her very well and she wanted to do it because of her personal circumstances but she didn't get the buy-in from her colleagues" (#2 service/admin lead)
**Monitoring and feedback**	Present	Adopters valued summative evaluation and describe their plans for how to do it (product performance, the broader effects of embedding the product and attractiveness, ecological effect, acceptability to staff and patients)	"[name of the evaluation lead] is doing the evaluation, so working with him to look at what are we trying to capture, what changes are we trying to show, what improvements might the system provide us." (#3 clinical lead)
**ADOPTERS (INCLUDING PATIENTS AS ADOPTERS AS WELL AS STAFF)**			
**Staff: learning style – accounted for**	Absent	One person felt their preferred learning (kinaesthetic/visual) style was not accounted for	"I feel like we did have some kind of talk through but actually I very much felt being that kind of kinaesthetic, visual learner that I needed to go on and actually use the software, but we can't because we can't get logged into it." (#4 clinical lead)
**Staff: values and goals - supportive**	Present	Involved adopters have vision of how to use the innovation, are flexible, risk-taking, and happy to experiment with it<br>All strongly believe in asynchronous consultations in general, but most expressed limited confidence in this specific system at the time, but with	"I think the other doctor selected a couple of us that are quite flexible in terms in how we practice in our profession" (#1 clinical lead)<br><br>"I think confident is a bit of a strong word, but I'm definitely interested and keen to see how they make it work, and if they can make it work." (#2 service/admin lead)

		time many change their mind	
**Staff: high motivation**	Present	All adopters are highly motivated<br>Concerns about resistance of other clinicians stated, and normalisation of new ways of working and good uptake by patients viewed as a mean to overcome it	"The clinicians themselves are very motivated to changing how they operate and engage with new digital tools, so they're highly motivated to do that and they know their domain very, very well." (#1 technical expert/lead)<br><br>"I think there needs to be a willingness. You know, a willingness of people and a desire... a desire to improve it and there absolutely is that." (#2 service/admin lead)
**Staff: social networks - supportive**	Present	Seeing similar innovation working elsewhere have inspired clinicians to adopt it in NHSG<br>Some reported seeing broader changes in the attitude of clinicians towards acceptance of asynchronous consultations as a new normal	"Recently, one of the consultants has said, "What's about these asynchronous clinics? That might be really good for the same clinic that I was sort of proposing it for." What I guess I've observed is that other people's opinions of technology in healthcare have changed too. Something that seemed absurd 18 months ago is actually seeming like a good idea now" (#4 clinical lead)
**Targeting and selecting patients (added by the authors)**	Present	Adopters reported much effort dedicated to identifying the right group of patients, considered in the process condition type, demographic characteristic, psychological factors, and lifestyle<br>Adopters held assumptions about who it may works and why, and while some reported those assumptions being challenged by reality, they continued being expressed	"Well, for that autoimmune hepatitis it was that particular condition [clinical lead] felt was suitable as the first try at this and she also thought she'd have a high uptake because of the demographic, because of the age range and the working nature of the folk with the condition she felt that they were ones who were likely to not want to come into clinic." (#1 technical expert/lead)<br><br>"I guess the people who haven't engaged with it are often people that don't have internet, they don't have email addresses. They either don't have internet or they've got internet but they don't have an email address. I guess some people just have a preference to speak to somebody, they don't want to complete online forms." (#3 clinical lead)

**Patients' engagement and strategies to supporting or induce it (added by the authors)**	Present	Strategies used by adopters to improve patient engagement included more convenient appointment booking process, information giving, using the term 'assessment' not 'appointment', compulsory online engagement before an asynchronous journey	"I think there's some challenges around language and things used there. So what we did was call it a digital assessment so it took away that notion of a specific time of a day to sort of encourage that flexibility." (#1 service/admin lead)<br><br>"The pandemic was just starting... had just started, it was a lot of uncertainty, so they were maybe a lot of information being thrown at them by media, by the medical care as well about Covid and everything, and maybe just a lack of understanding or being cold called and going, "Yeah, yeah, I'll go up for..." you know, "Sign up for that," and then when it came to actually using it... there was different, there was a variety of different levels of reasons why they just said no straightaway (#3 service/admin lead)<br><br>"We've done a lot of work in what the patient information leaflets were like, letters were like, when we're phoning the patients how we're explaining it to them. I think feedback from when patients did an onboard feedback on the reasons why they didn't onboard. We took that onboard and changed our information leaflets to change... simple things such as changing the wording." (#3 service/admin lead)<br><br>"There're other things in terms of us updating our external website in terms of information, looking at our patient information leaflet, our PIL in terms of updating that. You know, there's some things that we can do, and these are only things that I have identified" (#1 project management lead/expert)
**Patient involvement in the specification (added by the authors)**	Partially present	Helpful but limited to developer's user experience work, involvement of patients<br><br>One person saw that limited direct involvement as a problem<br><br>Patients indirectly influence how the system is run, for example, changes around	"It's a bit of a glaring admission that we don't have a patient on the set-up team, but I guess I'm going to kind of blame Covid because it was all stalled, and then it was relaunched again and we were on quite a tight timescale, and obviously we weren't really in a situation for bringing people in for that type of discussion" (#3 clinical lead)<br><br>"We've also got input from the patient around medication concerns, diagnostic concerns, and what they hope to achieve from coming to the pain service and any other sort of general concerns they might have." (#3 clinical lead)

		whom it’s communicated with patients or new functions added to the system (e.g., medication concerns)	
**ASSIMILATION BY THE SYSTEM**			
**Complex, nonlinear process - accounted for**	Absent	All staff found the process nonlinear, complex (often more than anticipated) and frustrating (they seem to weigh up efforts-benefits when deciding if it’s worthwhile)<br><br>Teams reported working hard around setbacks between development, implementation, and routinisation (detailed in Table 1)<br><br>Some adopters reflected on importance of timing and sequencing tasks (e.g., finding the sweet point for integration) and stakeholders’ involvement (e.g., not involving clinicians/patients too early/late). Lack of a national guidance on generic steps (in these critical/optional steps locally) was lacking.	"What else has been unexpected? Just how complex the process is, I guess." (#4 clinical lead)<br><br>““I think the area that frustrated us more was the integration side of things because we already had integration up and running with dermatology and from our perspective it should’ve been a relatively simple process” (#2 technical expert/lead)<br><br>“But the reality turned into that it was an absolute uphill battle to even get it live in one of the two identified areas” (#2 project management lead/expert)<br><br>“I think they could all relate to this story of it being given a task and trying to implement it by a date and not hitting that date, and then feeling that you’re constantly trying to push something up a hill just to try and get it done. I don’t think this is necessarily exclusive to this particular bit of work; it just happened to be the focus of the evaluation for us.” (#2 project management lead/expert)<br><br>“If you integrate it too early, you might end up with something that won't scale very well. If you integrate it too late, you might end up with lots of messy ways of working with lots of different workarounds that people don't want to change from. I think there is definitely a sweet point of when's the right sweet spot to integrate and I don't think we got that right with this one" (#1 technical expert/lead)

**IMPLEMENTATION AND ROUTINISATION**			
**Decision making devolved to frontline teams**	Partially present	Decision-making devolved to pain and gastroenterology adopters, but not to dermatology adopters<br>Adopters wished for greater involvement of admin staff in decisions from early in the process	"Involvement of the stakeholders, as I say, the secretarial staff would have been absolutely brilliant at pointing out where the omissions might happen, or kind of documentation issues might happen that we would never foresee." (#4 clinical lead)<br>"I think having had it [dermatology system] described as a pilot I think as a service we didn't feel that our feedback or our concerns around process and things was maybe being considered as I would expect had it" (#1 service/admin lead)
**Hands-on approach by leaders and management**	Present	A lack of management skills initially (dermatology only) and continuity in project management overall were reported<br>Staff wished there was someone who oversees the whole process (like the project board and the core operational project team); keeps all the parties involved and informed and keeps advancing the project<br>A person who acted as a project manager, after the new pathways went live, provided a detailed account of their hands-on approach	"I think we ended up going over the same things more than once just to get to where we wanted to be. I certainly don't think it helped having changes in the leads with it." (#2 admin/service lead)<br>"I'm still a little bit unclear about the project board that would have been around that, but certainly the core operational project team, it would be their drive and determination to try and see something through and getting key you know, knowing who to contact, getting the key people involved in the project like your clinical leads and things to help, say you know, "Where are we with this? What's happening? This needs to be going live." (#1 project management lead/expert)
**Dedicated resources**	Partially present	There are many internal stakeholders involved. Efficient staff involvement, with allocated time and resources for clinical and admin staff, outpatient clinic coordinator, project	"I suppose that probably has been a bit of a barrier, at times, again getting people to really, truly immerse theirself [sic] into it and give it a chance" (#2 project management lead/expert)<br>"I mean clinicians are never going to take this on as just an extra thing; it will always need us to allocate clinical time" (#1 service/admin lead)<br>"There was a new appointment made a number of months ago, which has...

		management, evaluation lead, health intelligence was viewed as needed<br><br>Importance of continuous funding was stressed, but a background of fiscal constraint within the NHSG is unclear<br><br>The pain clinic obtained additional funding from the Modernising Patient Pathways Programme to employ a pharmacist to run a medication clinic (an evolved function of the system)	actually, you can see it's paid dividends now in terms of the take-up of the figures in terms of the digital appointments as well, but that's kind of an admin support." (#1 project management lead/expert)<br><br>"I think that's the positive to take is that you can embed something into the organisation and upscale it and move it, but you need to have those champions and people that are pushing for it and really taking it through. I think that's where the project has benefited from having good clinical input and yes, I think probably the clinical input's been a very good aspect of it." (#2 project management lead/expert)<br><br>"If it was funded appropriately that would have maybe improved things as in funding for NHS staff and for NSS involvement. There was very little funding available within the NHS" (#2 technical expert/lead)<br><br>"But in the interim, we got some MPPP [Modernising Patient Pathways Programme] funding, and some winter funds, money from Scottish government, and we employed a pharmacist who then, along with the nurse, took up the information that we had and sort of put in place the medication clinic" (#3 clinical lead)
**Human resources issues - low degree of**	Absent	There are issues with reappointments/loss of personnel	"I mentioned that our integration developer left here to join them in the summer and that didn't help. It's good for them but not so great for us." (#3 technical expert/lead)
**Internal communication - good**	Present	Adopters recommended: regular meetings, open and clear two-way communication, including relevant people from the start and keeping them informed thought, records of communication, clearly outlined roles<br><br>Important issues with communication were reported at pre-implementation stage but	"I think yeah, just open, clear communication, there just really needs… and I suppose as a service we need to be confident that if I'm saying, "I can't commit to having three hours a week of my clinical time spent on patients' non-attendances", I suppose I have to be confident that that's being at least acknowledged." (#1 service/admin lead)<br><br>"It was limited in terms of the handover that I was given" (#1 project management lead/expert)<br><br>"I have tried to ensure that there's a communication line with the operational staff, is what I would say, because originally it was really about reporting issues... if there was any issues. I couldn't see how that was then being communicated back to me as the project manager if they'd any issues in terms of how it worked," (#1 project management lead/expert)

		the person acting as a project manager, after the new pathways went live, provided a detailed account of their effort to implement the kind of communication that adopters wanted	
**External collaboration**	Partially present	Adopters reported networking with other health boards and universities<br><br>Pain and gastroenterology are down as a proof of concept. In a full operational system contract, escalation routes, roles and dedicated time would be covered in a Service Level Agreement document	"Well, I think it may be different for the Asynchronous one because I don't think there is a contract set up with NSS regarding this initiative. The systems are down as proof of concept rather than full operational systems where you would want to have a service level agreement arranged with all the parties." (#3 technical expert/lead)<br><br>"I don't mean red tape, and I don't want to keep using the word governance because that's unfair, but the requirements and the criteria that the organisation needed to satisfy it to actually get these things rolled out then became a bit of a barrier to actually getting them rolled out, and there didn't appear to be a clear escalation route to help resolve that in a timely fashion". (#2 project management lead/expert)
**Reinvention or development – a high degree of**	Present	Transferability of the innovation across specialities was viewed as critical for routinisation but is currently viewed as not as simple as translating groundwork from one speciality to another<br><br>Despite challenges, the system has been deployed and is being used with varying levels of success in two new clinics with	"If I had to guess, probably three to five specialties live with a pathway or two and then that would be... would give you enough knowledge that you could integrate it generically but not have a huge number of complicated workarounds in place." (#1 technical expert/lead)<br><br>"We can understand that some of the other services uptake hasn't been quite as good, so our uptake has been really good" (#3 clinical lead)<br><br>"I think from what I can see there is different drivers to be offering the digital appointment, different cohorts of patients that, I suppose, were more, perhaps, immersed in pathways enabled by technology before. There were some that were quite familiar with the process of using technology or not having a face-to-face or a telephone call to engage with clinical staff." (#2 project management lead/expert)

		diverse patient populations and needs	"I do not see why in a properly selected group of patients or conditions could not be adopted in other areas. Like, it would be really good for patients with endocrine problems because the majority of their management is pretty standardised" (#1 clinical lead)
**Feedback on progress**	Present	Timely feedback (formative evaluation) on adopters' and patients' views welcome to make system adjustments before routinisation	"I think there just needs to be a good opportunity to feedback and evaluate and make changes rather than press ahead with something that we know is good but could be better if we were able to make amendments." (#1 service/admin lead)
**PERCEIVED CONSEQUENCES**			
**Perceived overall success of implementation**	Present	At the time of early phase interviews, there was a perceived sense of dermatology implementation not being successful (due to low perceived uptake), but reportedly that changed around the time the new pathways went live<br><br>During the phase interviews, adopters reported not straightforward but overall successful uptake (at the time) of pain pathways and one of the gastroenterology pathways.	"I think if we were able to spread further… so we will continue to use it and I think it will slowly grow, but we are struggling to get other clinicians to engage with it for the reasons that I've been through. So we will continue to use it but the confidence in the benefits isn't quite there at the moment." (#1 service/admin lead)<br><br>"I could see in February... was it February or March, there's quite a dramatic increase in Dermatology take-up in terms of the number of appointments that have went through. I'm attributing it to colleagues like that relief coordinator and colleagues in possible conversations that they're having with the clinicians." (#1 project management lead/expert)<br><br>"I suppose, in the face of all the adversity and challenges that the work has faced, the fact that that still is getting used and patients are still buying into shows that, I guess, as an attempt to introduce this and upscale it, it can be done [..] You know, it's a lot of very uncertain times, so I think just to even get a change enabled and live has been, that has been successful. You know that... something to be celebrated but I suppose the... yes, there's pros and cons to that, but the fact that it is up and running and, in these services, has been very good." (#2 project management lead/expert)<br><br>"I guess the uptake has been good so to some extent showing that's acceptable to patients and they have completed the form. There's been a couple of the forms that have not been fully completed, but on the whole,

			they've been fully completed, and the percentage uptake has been high [..] I think it's got a place for some patients, well for a lot of patients, but there needs to be an alternative option because it's not for everybody." (#3 clinical lead)

Table S3. Main setbacks to the deployment of pain and gastroenterology systems.

Delay type with details	Illustrative quotes from staff
**Delay for contractual reasons (4 months):**<br>– Innovation and transformation teams misused time going through the same things<br>– Delays in procurement led to a window of opportunity being missed with National Service Scotland (NSS), and the project getting deprioritised	*"The agreement for the emergency procurement with finance and the senior leadership team had been made when it was the innovation folk who were leading it. But then when it moved over to be the transformation folk, the transformation people seemed to have to go through a lot of the same loops again so seemed to have to go back to our senior leadership team and seemed to have to go then back to the finance director and go back through some of the things that I thought had already been ironed out." (#1 technical expert/lead)*<br>*"I think NHS Grampian didn't act upon the emergency procurement process rapidly enough for us to get the window with NSS when they had the capacity to do it, as I look back." (#1 technical expert/lead)*
**Delay for technical reasons (6 months):**<br>– The system architecture not being in place<br>– NSS availability was a large delay over summer of 2021 (capacity issues preventing it from being done)<br>– NHSG eHealth team lost critical personnel (capacity issue)<br>– User acceptance test (UAT) has taken longer than expected	*"Actually, in reality it's a technical issue to undertake some mapping work. I mean I guess I would say without the integration the service would've been live about six months ago. Well maybe not six, maybe three/four months ago, that's probably a bit much." (#2 technical expert/lead)*<br>*"It's really been the bottleneck at NSS that's caused a lot of delays, and then also some personnel changes at NSS which have resulted in the people who've got knowledge of how that integration works moving on to new positions" (#2 technical expert/lead)*
**Delays exacerbated by delays in scheduling the integration work and the subsequent testing (1 month):**<br>– Change of a project lead<br>– Troubleshooting one issue that has arisen during testing (integration and the PDF appointment outcome message reaching NHS Grampian)<br>– An issue has been identified with the system penetration (PEN) testing (process and visibility of tasks needing done/coordinated)<br>– Getting the governance documents approved and signed off (capacity issue preventing this from being looked at / taking priority)	*"There's been a bit of glitch. Yes, it's all sort of stopped because there was a change in the project lead and we had this problem with it sitting with IG for whatever that reason is" (#1 technical expert/lead)*<br>*"There is another interface as well which they need to do which is once the patient journey has been completed and there is a summary document created, that is sent from the Asynchronous application via NSS to NHS Grampian Integration and the summary document is uploaded into our electronic patient record software which is SCI Store. That is the two integrations that are required that involves me anyway." (#3 technical expert/lead)*

PDF - a PDF summary of the appointment that goes into SCI Store and GP Practice systems; **PEN testing** – a simulated cyber-attack against your computer system to check for exploitable vulnerabilities; **UAT-** a type of testing performed by the end-user or the client to verify/accept the software system before moving the software application to the production environment.

Figure S1. Comparison of the distribution of deprivation in the Grampian region and those who chose to use the asynchronous consultation service.

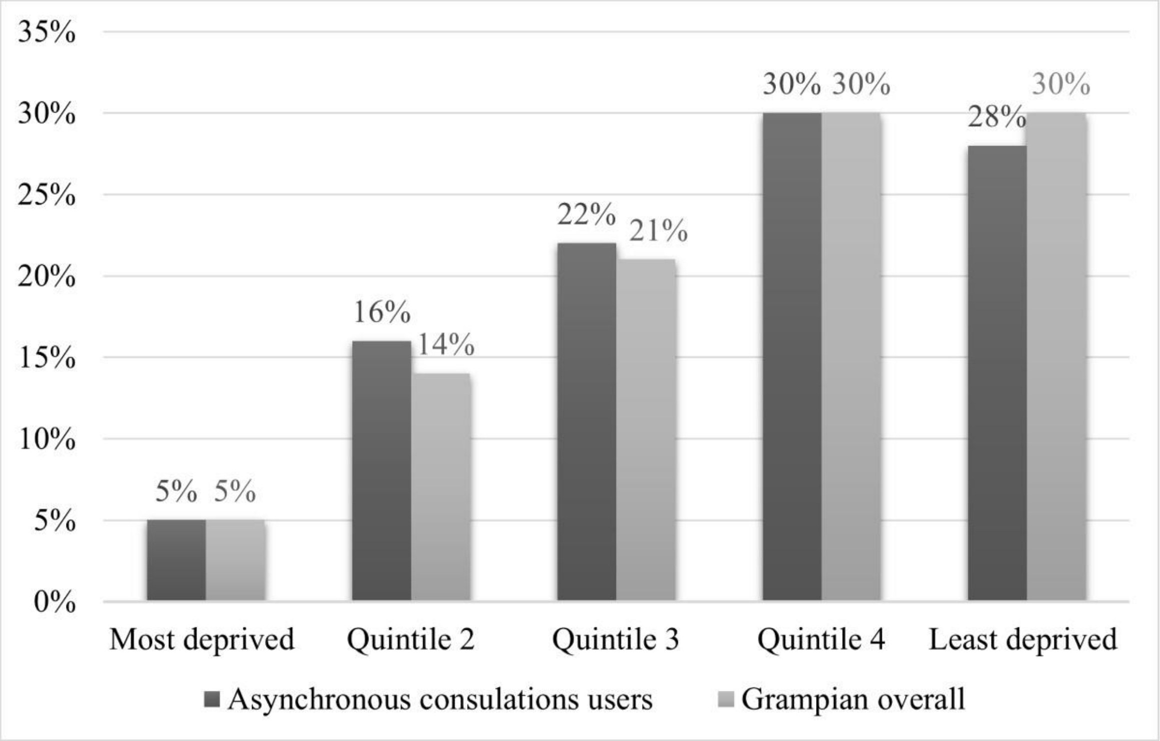

Supplement: Multimedia Appendix 2 [file jmir_v26i1e48092_app2.pdf]
